# SNAI1 is a prognostic biomarker and correlated with immune infiltrates in gastrointestinal cancers

**DOI:** 10.18632/aging.103667

**Published:** 2020-08-21

**Authors:** Jiaying Fang, Zan Ding

**Affiliations:** 1 The Institute of Metabolic Diseases, Baoan Central Hospital of Shenzhen, The Fifth Affiliated Hospital of Shenzhen University, Shenzhen 518102, Guangdong, China; 2 Medical Department, Affiliated Huadu Hospital, Southern Medical University (People’s Hospital of Huadu), Shenzhen 518102, Guangdong, China

**Keywords:** gastrointestinal cancers, EMT, immune infiltration, biomarker

## Abstract

Epithelial-mesenchymal transition (EMT)-related genes play an important role in immunosuppression. However, the correlations of EMT-related genes to prognosis and tumor-infiltrating lymphocytes in different cancers remain unclear. TCGA, GEO databases were used to analyze the expression, prognosis, and immune infiltration of EMT markers in cancer. RT-qPCR, immunohistochemistry, and western blot were used to analysis the expression and prognosis of SNAI1 in gastrointestinal cancers. High SNAI1 expression was closely related with poorer overall survival in gastrointestinal cancers in TCGA cohort. High SNAI1 expression was closely related with poorer overall survival in gastrointestinal cancers, and was validated in GEO database. Simultaneously, high expression of SNAI1 correlates with clinical relevance of gastric cancer. Moreover, SNAI1 expression was associated with tumor-infiltrating immune cells in gastrointestinal cancers. In addition, RT-qPCR, immunohistochemistry, and western blot showed SNAI1 expression was higher in gastrointestinal cancers compared to the normal tissues. Finally, high SNAI1 expression was closely related with poorer overall survival and correlates with clinical relevance of gastrointestinal cancers in an independent validation cohort. In summary, the results approaches to suggest that SNAI1 can be used as a prognostic biomarker for determining prognosis and immune infiltration in gastrointestinal cancers.

## INTRODUCTION

Invasion and metastasis are the important characteristics of gastrointestinal (GI) cancers, and leads to a poor prognosis [1, 2]. Surgery, radiotherapy, and chemotherapy are the predominant treatments for GI [3, 4]. Although comprehensive treatment may cure some patients with early-stage GI, most patients are diagnosed with advanced stage [5]. With the rapid development of medical immunology and molecular biology techniques, immunotherapy as a new treatment method has received extensive attention in the field of cancer therapy [6]. Immunotherapy involves destroying tumor cells by activating and training the patient’s immune system to recognize tumor cells as targets [7, 8]. However, only 10% to 20% of the population can benefit from immunotherapy [9, 10]. Due to the heterogeneity of tumors, the current biomarkers for predicting prognosis have certain limitations. Therefore, this field requires new biomarkers as prognostic indicators to effectively enhance prognosis and individualized treatment.

Epithelial-mesenchymal transition (EMT) is a multistage process in which epithelial cells develop into mesenchymal-like cells with a large number of distinct genetic and epigenetic alterations [11]. EMT also occurs in cancer, which endows invasive, metastatic, and immunosuppressive properties upon cancer cells that favor successful colonization of distal target organs [12]. The process of EMT is regulated by a variety of cytokines and transcription factors, among which the changes of migration and invasion are the most important characteristics of EMT, and the changes of EMT-related genes expression are the key to the formation of EMT [13]. These findings suggest the EMT-related genes play an important role in cancer progression, invasion, and metastasis.

This study focused on six key EMT-related genes (CDH1 encoding E-cadherin, CDH2 encoding N-cadherin, EMT-induced transcription factor: SNAI1, SNAI2, TWIST1, and VIM) [14]. Firstly, we detected the expression of EMT-related genes in human cancers. Secondly, we comprehensively analyze EMT-related genes correlation with prognosis of cancer and was validated in GEO database. Moreover, we detected the relationship of SNAI1 and tumor-infiltrating immune cells in gastrointestinal cancers. Finally, we used RT-qPCR, immunohistochemistry, and western blot to detect the expression of SNAI1 in gastrointestinal cancers and paired adjacent normal tissue, and SNAI1 was validated as a marker in an independent gastrointestinal cancers validation sample cohort.

## RESULTS

### The six EMT-related genes expression in human cancers

**Figure 1:**
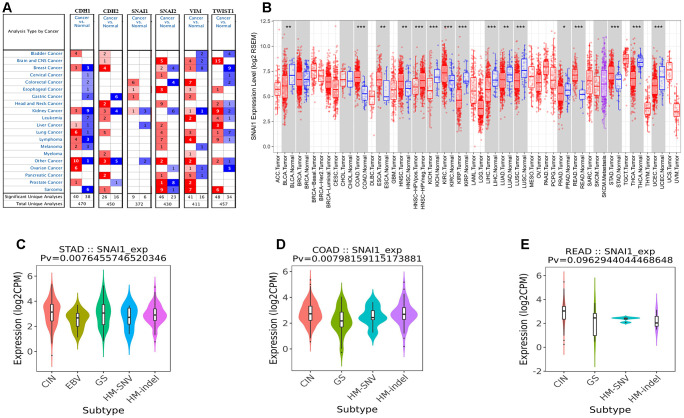
**The six EMT markers expression levels in different types of human cancers.** (**A**) Increased or decreased six EMT markers in datasets of different cancers compared with normal tissues in the Oncomine database. (**B**) SNAI1 expression levels in different tumor types from TCGA database were determined by TIMER (*P < 0.05, **P <0.01, ***P < 0.001). (**C**–**E**) Correlation of SNAI1 expression and immune subtypes (wound healing, IFN-gamma dominant, inflammatory, lymphocyte depleted, TGF-β dominant) in COAD (colon adenocarcinoma), READ (rectum adenocarcinoma), and STAD (stomach adenocarcinoma).

To analysis the expression of EMT-related genes in human cancers, the Oncomine database was used to analysis the EMT-related genes mRNA levels in human cancers. Specifically, as shown in Figure 1A, the SNAI1 expression was higher in colorectal cancer, esophageal cancer, gastric cancer, and kidney cancer. To further analysis EMT markers in human cancers, we compare the expression level of EMT-related genes in TCGA dataset (Supplementary Figure 1A–1E). Specifically, as shown in Figure 1B, compared with normal tissues, the SNAI1 expression was significantly higher in bladder urothelial carcinoma (BLCA), colon adenocarcinoma (COAD), esophageal carcinoma (ESCA), head and neck squamous cell carcinoma (HNSC), kidney chromophobe (KICH), kidney renal clear cell carcinoma (KIRC), rectum adenocarcinoma (READ), stomach adenocarcinoma (STAD). However, SNAI1 expression was significantly lower in kidney renal papillary cell carcinoma (KIRP), liver hepatocellular carcinoma (LIHC), lung adenocarcinoma (LUAD), lung squamous cell carcinoma (LUSC), Prostate adenocarcinoma (PRAD), thyroid carcinoma (THCA), corpus Endometrial Carcinoma (UCEC).

### Survival analysis of six EMT-related genes in cancers

**Figure 2:**
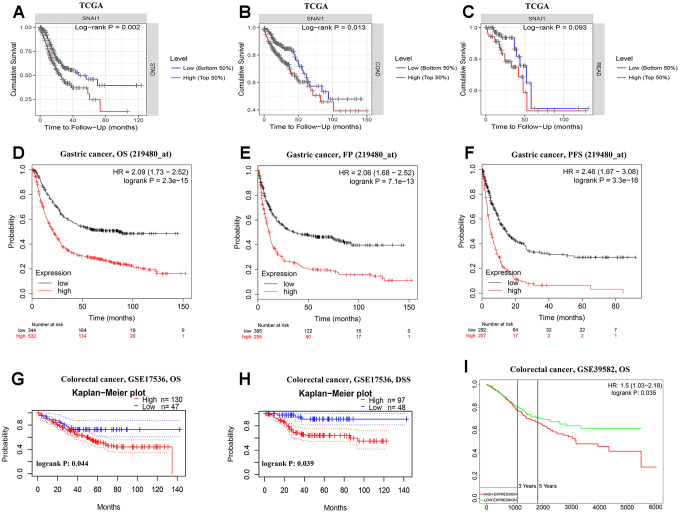
**Kaplan-Meier survival curves comparing the high and low expression of SNAI1 in gastrointestinal cancers in the TCGA dataset and GEO dataset.** (**A**–**C**) Survival curves of overall survival in COAD (colon adenocarcinoma), READ (rectum adenocarcinoma), and STAD (stomach adenocarcinoma) in TCGA cohorts. (**D**–**F**) Survival curves of OS, FP, and PFS in six STAD cohorts (GSE29013, GSE31210, GSE31908, GSE43580, GSE50081, GSE8894). (**G**–**I**) Survival curves of OS and DSS in two CRC cohorts (GSE17536, GSE39582).

Next, to inspect whether six EMT-related genes were related with prognosis in cancer patients, GEPIA site was used to analysis the prognosis of genes in cancers by using the TCGA dataset (Supplementary Figures 2–7). Notably, high SNAI1 expression levels was closely related with poorer prognosis of overall survival (OS) in STAD (*p*=0.002), COAD (*p*=0.013), ESCA (*p*=0.031), KIRP (*p*=0.045), LGG (*p*<0.001), LUAD (*p*=0.026), LUSC (*p*<0.001), MESO (*p*=0.007), OV (*p*=0.048), and THYM (*p*=0.001) (Figure 2A, 2B and Supplementary Figure 4). Simultaneously, high SNAI1 expression levels was moderate closely related with poorer prognosis of OS in READ (*p*=0.093) (Figure 2C). Therefore, we focus on SNAI1 expression and prognosis in gastrointestinal cancers.

Next, to inspect whether SNAI1 expression was related with subtype of gastrointestinal cancers, we divide gastrointestinal cancers into five subtypes (CIN, EBV, HM-SNV, HM-indel) [15]. We found high SNAI1 expression was significantly related with subtype of STAD (*p*=0.008) and COAD (*p*=0.008), and moderate closely related with READ (*p*=0.096) (Figure 1C–1E). To validate the prognostic potential of SNAI1 in STAD, we used Kaplan-Meier plotter database to validate the prognostic potential of SNAI1 in STAD. Interestingly, a cohorts including GSE62254, GSE14210, GSE15459, GSE22377, GSE29272, and GSE51105 indicated that high SNAI1 expression was closely related with poorer prognosis in STAD (OS HR = 2.09, 95% CI =1.73 to 2.52, *P*<0.001; FP HR = 2.06, 95% CI =1.68 to 2.52, *P*<0.001; PFS HR = 2.46, 95% CI = 1.97 to 3.08, *P*<0.001) (Figure 2D–2F). Moreover, two cohorts including GSE17536, GSE39582 indicated that high SNAI1 expression was closely related with poorer prognosis in CRC (OS HR = 1.50, 95% CI =1.03 to 2.18, *P*=0.035) (Figure 2G–2I).

### High expression of SNAI1 correlates with clinical relevance of STAD

Next, we examined the associationship between the SNAI1 expression and clinical relevance of STAD. As shown in Table 1, high SNAI1 expression was closely related with poorer prognosis in female (OS HR = 2.24, *P*<0.001; PFS HR =1.91, *P* = 0.002) and male (OS HR = 2.27, *P* <0.001; PFS HR =2.43, *P*<0.001). Moreover, high SNAI1 expression was closely related with poorer OS and PFS in stage 1 (OS HR = 3.63, *P*=0.011; PFS HR =2.88, *P* = 0.023), stage 2 (OS HR = 2.03, *P*=0.021; PFS HR =1.93, *P* = 0.030), stage 3 (OS HR = 1.98, *P*<0.001; PFS HR =2.06, *P* <0.001), and stage 4 (OS HR = 2.06, *P*<0.001; PFS HR =2.30, *P* <0.001) of STAD, and poorer OS and PFS in TNM stage. Furthermore, high SNAI1 expression was closely related with poorer prognosis in the lauren classification (OS HR = 1.89, *P*<0.001; PFS HR =2.33, *P* <0.001), moderate differentiation (OS HR = 1.94, *P*=0.046; PFS HR =2.27, *P* =0.011), negative (OS HR = 1.98, *P*<0.001; PFS HR =2.02, *P* <0.001) and positive (OS HR = 2.14, *P*<0.001; PFS HR =2.36, *P* <0.001) HER-2 status (Table 1). These results suggest that high SNAI1 expression can impact the prognosis in STAD with lymph node metastasis, and SNAI1 is an independent prognostic marker but can also predict the clinicopathological features of STAD.

**Table 1:** Correlation of SNAI1 mRNA expression and clinical prognosis in gastric cancer with different clinicopathological factors by Kaplan-Meier plotter (GSE62254, GSE14210, GSE15459, GSE22377, GSE29272, GSE51105).

**Clinicopathological characteristics**	**Overall survival (n = 882)**			**Progression-free survival (n = 646)**		
	**N**	**Hazard ratio**	**P**	**N**	**Hazard ratio**	**P**
**SEX**						
Female	244	2.24 (1.46–3.35)	1.0e-04	201	1.91 (1.25–2.91)	0.002
Male	567	2.27 (1.81–2.83)	1.5e-13	438	2.43 (1.90–3.11)	2.7e-13
**STAGE**						
1	69	3.63 (1.26–10.46)	0.011	60	2.88 (0.78–10.63)	0.023
2	145	2.03 (1.10–3.74)	0.021	131	1.93 (1.05–3.55)	0.030
3	319	1.98 (1.42–2.76)	3.6e-05	186	2.06 (1.38–3.07)	3.2e-05
4	152	2.06 (1.40–3.03)	2.0e-04	141	2.30 (1.54–3.43)	2.9e-05
**STAGE T**						
2	253	2.18 (1.42–3.34)	2.6e-05	239	1.93(1.27–2.92)	0.002
3	208	2.06 (1.40–3.04)	1.7e-04	204	1.80 (1.23–2.63)	0.002
4	39	2.40 (1.03–5.58)	0.036	39	5.66 (2.22–14.41)	5.5e-05
**STAGE N**						
0	76	2.80 (1.17–6.70)	0.016	72	2.63 (1.11–6.24)	0.023
1	232	2.45 (1.61–3.71)	1.3e-05	222	2.12 (1.44–3.14)	1.2e-05
2	129	2.67 (1.66–4.31)	2.7e-05	125	2.44 (1.54–3.84)	7.9e-05
3	76	2.76 (1.59–4.79)	1.8e-05	76	2.38 (1.38–4.10)	0.001
1+2+3	437	2.15 (1.65–2.80)	7.9e-09	423	1.90 (1.47–2.45)	5.0e-07
**STAGE M**						
0	459	2.09 (1.58–2.76)	1.5e-07	443	1.85 (1.41–2.41)	4.6e-06
1	58	2.13 (1.11–4.08)	0.020	56	1.81 (0.97–3.36)	0.059
**LAUREN CLASSIFICATION**						
Intestinal	336	2.78 (2.02–3.82)	5.0e-11	263	2.32 (1.61–3.34)	3.3e-06
Diffuse	248	1.89 (1.34–2.65)	2.0e-04	231	2.33 (1.47–3.69)	2.0e-04
**DIFFERENTIATION**						
Poor	166	1.25 (0.84–1.87)	0.277	121	1.60 (1.02–2.53)	0.041
Moderate	67	1.94 (1.00–3.75)	0.046	67	2.27 (1.19–4.34)	0.011
**PERFORATION**						
No	169	1.70 (1.13–2.58)	0.011	58	2.35 (1.20–4.62)	0.011
**TREATMENT**						
Surgery alone	393	1.71 (1.28–2.28)	2.5e-05	375	1.51 (1.14–2.00)	0.004
5 FU based adjuvant	158	1.66 (1.13–2.45)	0.009	153	1.68 (1.14–2.48)	0.008
Other adjuvant	80	2.55 (1.02–6.40)	0.039	80	2.34 (1.01–5.43)	0.042
**HER2 STATUS**						
Negative	641	1.98 (1.58–2.49)	2.0e-09	408	2.02 (1.54–2.65)	2.2e-07
Positive	425	2.14 (1.57–2.91)	7.7e-07	233	2.36 (1.65–3.39)	1.4e-06

### SNAI1 expression was correlated with tumor-infiltrating lymphocytes (TILs)

**Figure 3:**
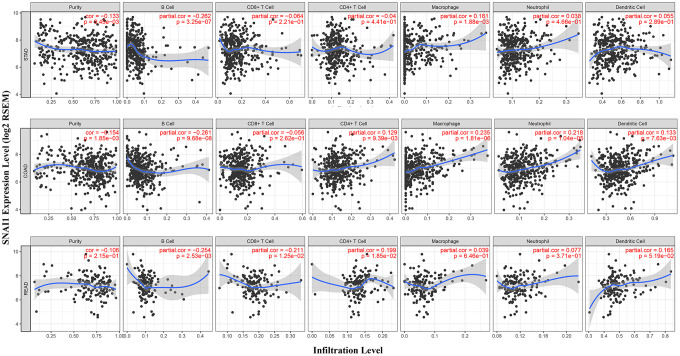
**Correlation of SNAI1 expression with immune infiltration level in COAD (colon adenocarcinoma), READ (rectum adenocarcinoma), and STAD (stomach adenocarcinoma).** (**A**) SNAI1 expression is significantly negatively related to tumor purity and infiltrating levels of B cells and has significant positive correlations with infiltrating levels of macrophages in STAD, other than CD8+ T cells, CD4+ T cell, neutrophils, and dendritic cells. (**B**) SNAI1 expression is significantly negatively related to tumor purity and infiltrating levels of B cells and has significant positive correlations with infiltrating levels of CD4+ T cell, macrophages, neutrophils, and dendritic cells in COAD, other than CD8+ T cells. (**C**) SNAI1 expression has no significant correlations with tumor purity, macrophages, and neutrophils in READ.

TILs are an independent predictor in cancers [16, 17]. Therefore, TIMER database was used to infer the relations between abundance of TILs and expression of SNAI1. The relationship between SNAI1 expression and TILs in different types of cancer was shown in Supplementary Figure 8. Specifically, SNAI1 expression was negative closely related with infiltrating levels of tumor purity and B cell in STAD, and positively closely related with macrophages in STAD. Moreover, SNAI1 expression was negative closely related with infiltrating levels of tumor purity and B cell in COAD, and positively closely related with CD4+T cells, macrophages, dendritic cells, and neutrophils in COAD. Finally, SNAI1 expression was negative closely related with infiltrating levels of B cell and CD8+ T cells in READ, and positively closely related with CD4+T cells and dendritic cells in READ (Figure 3A–3C).

**Figure 4:**
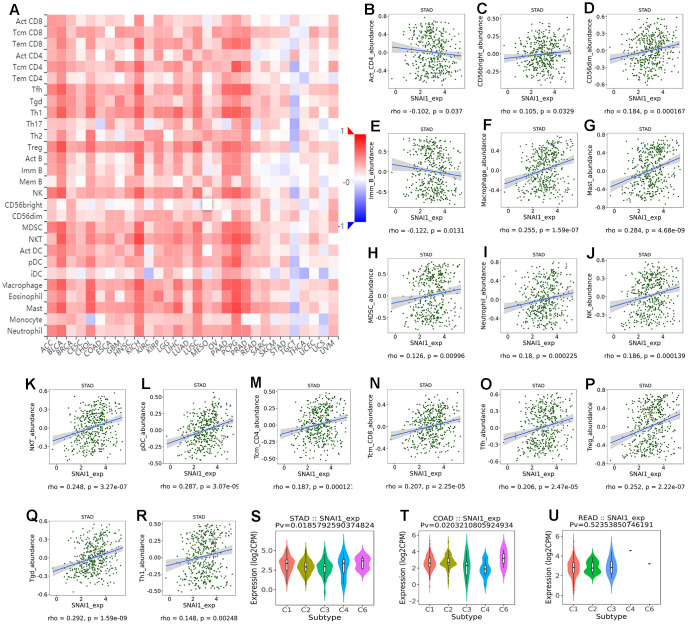
**Correlation of SNAI1 expression with immune cells in cancer.** (**A**) The landscape of relationship between SNAI1 expression and TILs in different types of cancer(red is positive correlated and blue is negative correlated). (**B**–**R**) SNAI1 expression was positively closely related with infiltrating levels of CD56bright, CD56dim, macrophage, mast, MDSC, neutrophil, NK, NKT, pDC, tcm_CD4, tcm_CD8, Tfh, Tgd, Th1, Treg in stomach cancer, and was negatively correlated with infiltrating levels of act_CD4, Imm_B in stomach cancer. (**S**–**U**) Correlation of SNAI1 expression and immune subtypes (wound healing, IFN-gamma dominant, inflammatory, lymphocyte depleted, TGF-β dominant) in COAD (colon adenocarcinoma), READ (rectum adenocarcinoma), and STAD (stomach adenocarcinoma).

Next, to examine which TILs was related with gastrointestinal cancers, TISIDB database was used to infer the relations between abundance of 28 TILs and expression of SNAI1. The landscape of relationship between SNAI1 expression and TILs in different types of cancer was shown in Figure 4A. The relations between abundance of 28 TIL types and expression of SNAI1 in gastrointestinal cancers was significant correlated (Figure 4B–4R and Supplementary Figure 9). Next, we detected the associations between SNAI1 expression and immune subtypes in gastrointestinal cancers, and we divided the cells into six immunophenotypes (C1: wound healing, C2: IFN-gamma dominant, C3: inflammatory, C4: lymphocyte depleted, C5: immunologically quiet, C6: TGF-β dominant) [20]. Specifically, SNAI1 expression was correlated with immune subtypes (wound healing, IFN-gamma dominant, inflammatory, lymphocyte depleted, TGF-β dominant) in STAD and COAD, but not in READ (Figure 4S–4U). These results suggest that SNAI1 expression was associated with tumor-infiltrating immune cells in gastrointestinal cancers.

### Validation SNAI1 in gastrointestinal cancers

**Figure 5:**
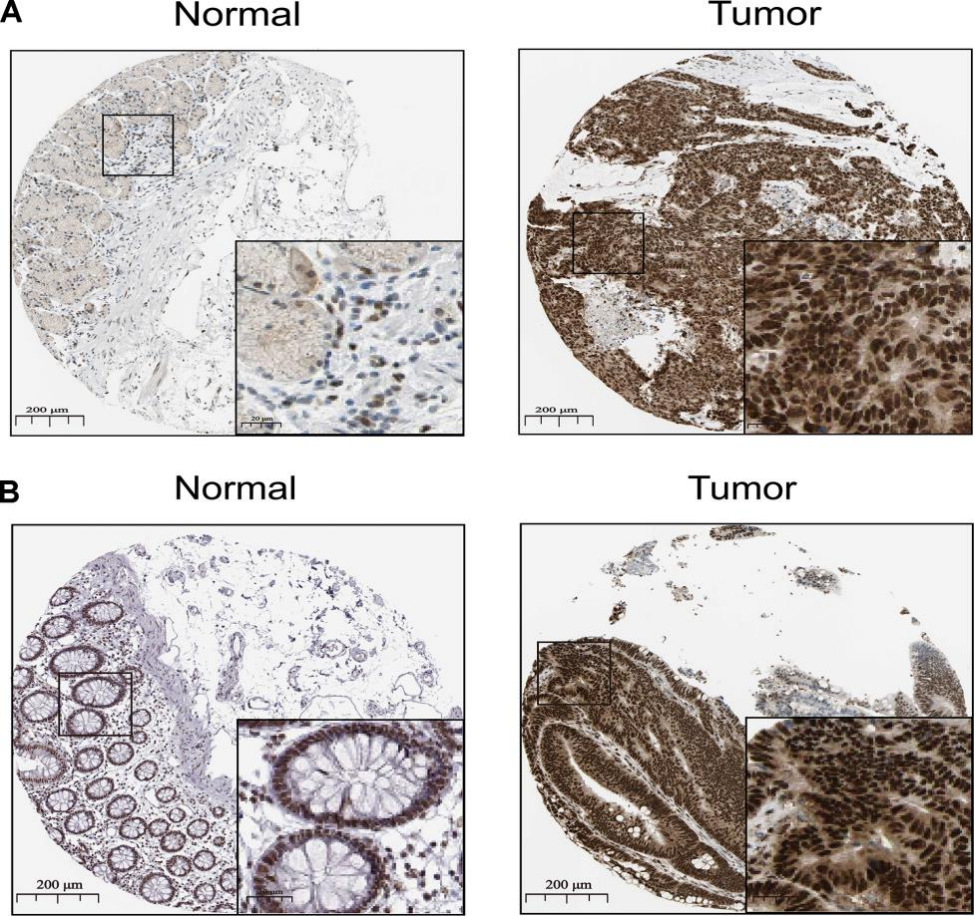
**The distribution of SNAI1 in cancer.** (**A**, **B**) Representative IHC images of SNAI1 expression in normal stomach tissues, stomach cancer tissues(A), normal tissues, and colorectal cancer tissues (**B**).

**Figure 6:**
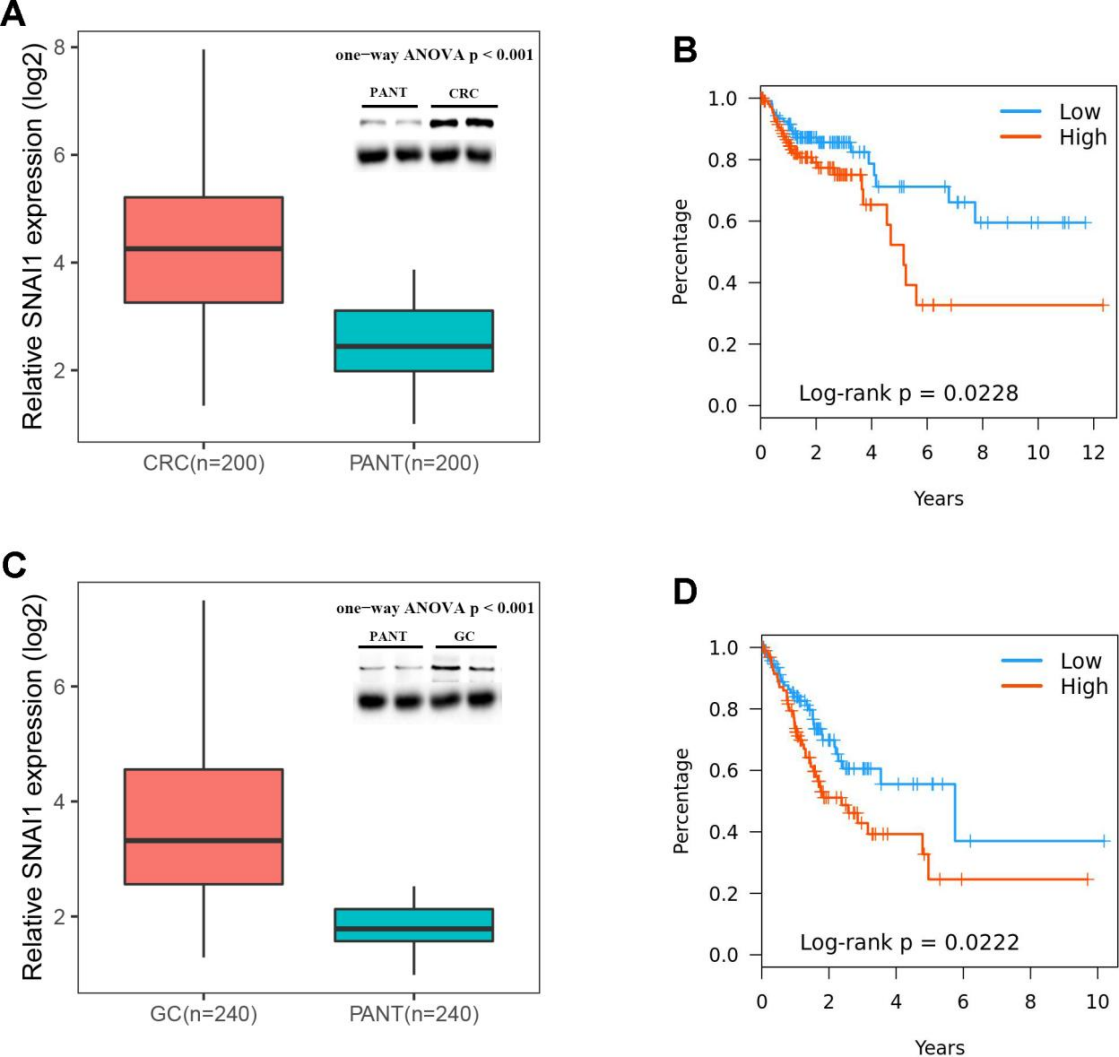
**Expressions of SNAI1 is positive correlated in CRC (colorectal cancer) and GC (gastric cancer).** (**A**) SNAI1 mRNA and protein levels are shown for the CRC and paired adjacent normal tissue (PANT). (**B**) Kaplan–Meier analysis of overall survival based on SNAI1 mRNA levels in 200 cases of CRC patients. (**C**) SNAI1 mRNA and protein levels are shown for the GC and paired adjacent normal tissue (PANT). (**D**) Kaplan–Meier analysis of overall survival based on SNAI1 mRNA levels in 240 cases of GC patients.

To further validate SNAI1 in gastrointestinal cancers, RT-qPCR was used to detect the SNAI1 mRNA expression in STAD, CRC, and paired adjacent normal tissue (PANT). Compared with the PANT group, the SNAI1 mRNA level was significantly higher in the CRC and GC group (Figure 6A, 6C). Next, we measured the SNAI1 protein level by immunohistochemistry and western blot, and the result showed compared with the PANT group, the SNAI1 level was significantly higher in the CRC and GC group (Figures 5A, 5B and 6A, 6C). Additionally, Kaplan–Meier and Cox’s proportional hazards regression model survival analysis revealed that patients with high expression levels of SNAI1 had shorter overall survival in CRC (HR = 1.71, 95% CI = 1.28 to 2.94, *P* = 0.023) and GC (HR = 1.68, 95% CI = 1.23 to 2.57, *P* = 0.022) (Figure 6B, 6D). Simultaneously, high expression of SNAI1 correlates with clinical relevance of CRC and GC (Table 2). To verify the relationship between SNAI1 and the diverse immune infiltrating cells, we focused on the correlations between SNAI1 and immune marker sets of various immune cells in gastrointestinal cancers. We analyzed the correlations between SNAI1 expression and immune marker genes of different immune cells, included CD8+ T cell, T cell (general), B cell, monocyte, tumor-associated macrophage (TAM), M1 macrophage, M2 macrophage, neutrophils, natural killer cell, dendritic cell, T helper cell (Th) 1, Th2, follicular helper T cell (Tfh), Th17, regulatory T cell (Treg), and T cell exhaustion. It is only when SNAI1 is related to all the markers of an immune cell that we think that SNAI1 is related to immune cells. The results revealed the SNAI1 expression level was significantly correlated with M2 macrophage in STAD, and was significantly correlated with monocyte, TAM, M2 macrophage, dendritic cell, Th1, and treg in CRC (Table 3). Correlation results between SNAI1 and TILsare similar to those in TCGA database (Figure 4B–4R and Supplementary Figure 9). These findings suggest that SNAI1 may regulate M2 macrophage in gastrointestinal cancers.

**Table 2:** Univariate and multivariate analyses of clinicopathological characteristics, SNAI1 with overall survival in independent validation cohort.

**Parameters**	**Univariate analysis^a^**		**Multivariate analysis^b^**	
	**HR (95% Cl)**	***P* value**	**HR (95% Cl)**	**P value**
**CRC (n=200)**				
Gender(male vs. female)	1.024 (0.681–1.547)	0.925	NA	NA
Age (y, >60 vs ≤60)	1.192 (0.835–1.927)	0.346	NA	NA
TNM stage (III and IV vs. I and II)	*2.101(1.218–3.709)*	*0.002*	*1.914(1.165–3.226)*	*0.009*
Pathologic M stage (M1 vs. M0)	*3.127(1.852–4.467)*	*0.003*	*2.519(1.248–3.814)*	*0.024*
Vascular invasion (negative vs. positive)	*2.300(1.113–3.671)*	*0.014*	1.562(0.779–3.530)	0.136
SNAI1 (≥median vs. < median)	*1.708 (1.276–2.937)*	*0.023*	*1.449 (1.091–2.279)*	*0.038*
**GC (n=240)**				
Gender(male vs. female)	1.447 (0.919–2.152)	0.453	NA	NA
Age (y, >60 vs ≤60)	1.237 (0.797–2.294)	0.674	NA	NA
TNM stage (III and IV vs. I and II)	*1.669(1.192–2.547)*	*<0.001*	*1.436(1.023–2.067)*	*0.007*
Pathologic M stage (M1 vs. M0)	*1.514 (1.038–2.027)*	*<0.001*	*1.336 (1.023–1.933)*	*0.012*
Vascular invasion (negative vs. positive)	*1.742 (1.228–2.368)*	*<0.001*	*1.502 (1.043–2.388)*	*0.008*
SNAI1 (≥median vs. < median)	*1.680 (1.234–2.574)*	*0.022*	*1.581 (1.126–2.256)*	*0.038*

^**a**^The data were subjected to Cox’s proportional hazards regression model. Bold italics indicate statistically significant values (P < 0.05),

^b^Multivariate analysis used stepwise addition and removal of clinical covariates found to be associated with survival in univariate models (P < 0.05) and final models include only those covariates that were significantly associated with survival (Wald statistic, P < 0.05). Bold italics indicate statistically significant values (P < 0.05).

**Table 3:** Correlation analysis between SNAI1 expression and markers expression of immune cells.

**Immune cells**	**Gene markers**	**STAD**		**CRC**	
		**Cor**	***P***	**Cor**	***P***
**CD8+ T cell**	CD8A	-0.084	0.091	0.171	*
	CD8B	0.163	***	0.065	0.321
**T cell (general)**	CD3D	-0.122	0.017	0.154	0.014
	CD3E	-0.089	0.071	0.210	***
	CD2	-0.089	0.074	0.221	***
**B cell**	CD19	-0.072	0.152	0.111	0.060
	CD79A	-0.094	0.059	0.148	0.025
**Monocyte**	CD86	0.124	0.018	0.500	***
	CD115 (CSF1R)	0.192	***	0.473	***
**TAM**	CCL2	0.300	***	0.573	***
	CD68	0.132	0.038	0.451	***
	IL10	-0.028	0.580	0.510	***
**M1 Macrophage**	INOS (NOS2)	-0.056	0.260	-0.141	0.019
	IRF5	0.052	0.300	0.170	*
	COX2(PTGS2)	0.200	***	0.231	***
**M2 Macrophage**	CD163	0.243	***	0.423	***
	VSIG4	0.222	***	0.470	***
	MS4A4A	0.141	***	0.480	***
**Neutrophils**	CD66b (CEACAM8)	0.140	***	0.052	0.392
	CD11b (ITGAM)	0.089	0.071	0.481	***
	CCR7	-0.024	0.632	0.200	***
**Natural killer cell**	KIR2DL1	-0.025	0.621	0.172	*
	KIR2DL3	-0.013	0.800	0.160	*
	KIR2DL4	-0.046	0.354	0.110	0.064
	KIR3DL1	-0.058	0.244	0.086	0.161
	KIR3DL2	-0.052	0.288	0.158	*
	KIR3DL3	0.002	0.971	0.053	0.382
	KIR2DS4	0.091	0.134	0.185	0.113
**Dendritic cell**	HLA-DPB1	-0.054	0.276	0.300	***
	HLA-DQB1	-0.082	0.098	0.183	**
	HLA-DRA	-0.091	0.068	0.322	***
	HLA-DPA1	-0.053	0.284	0.293	***
	BDCA-1(CD1C)	-0.018	0.722	0.211	***
	BDCA-4(NRP1)	0.366	***	0.561	***
	CD11c (ITGAX)	0.143	*	0.454	***
**Th1**	T-bet (TBX21)	-0.053	0.281	0.221	***
	STAT4	-0.034	0.500	0.264	***
	STAT1	0.029	0.562	0.264	***
	IFN-γ (IFNG)	-0.074	0.133	0.233	***
	TNF-α (TNF)	0.243	***	0.389	***
**Th2**	GATA3	0.017	0.742	0.367	***
	STAT6	-0.045	0.364	-0.036	0.557
	STAT5A	0.074	0.133	0.242	***
	IL13	0.098	0.049	0.240	***
**Tfh**	BCL6	0.194	***	0.499	***
	IL21	-0.110	0.032	0.123	0.053
**Th17**	STAT3	0.200	***	0.261	***
	IL17A	0.017	0.738	-0.153	0.015
**Treg**	FOXP3	0.058	0.244	0.364	***
	CCR8	0.063	0.223	0.383	***
	STAT5B	0.144	*	0.345	***
	TGFβ (TGFB1)	0.444	***	0.566	***
**T cell exhaustion**	PD-1 (PDCD1)	-0.003	0.959	0.205	***
	LAG3	-0.066	0.183	0.072	0.232
	CTLA4	0.010	0.831	0.300	***
	TIM-3 (HAVCR2)	0.087	0.081	0.474	***
	GZMB	-0.052	0.304	-0.02	0.757

STAD: Stomach adenocarcinoma; CRC: Colorectal cancer; TAM: Tumor-associated macrophage; Th: T helper cell; Tfh: Follicular helper T cell; Treg: Regulatory T cell; Cor, R value of Spearman’s correlation; *P < 0.01; **P < 0.001; ***P < 0.0001.

## DISCUSSION

Due to advances in treatment, the mortality rate of tumors has been declining in recent years, a large part of which is due to immunotherapy. Immunotherapy represented by anti-PD-1/PD-L1 monoclonal antibody drugs and CAR-T cell therapy has attracted much attention, and encouraging results have continued. Both of them are essentially the ability of human autoimmune system to recruit and activate human core immune guardian-T cells to identify and clear cancer cells through antigen-antibody response [18]. However, not every patient responds to this treatment, especially in gastrointestinal cancers [19]. Therefore, there is an urgent need to clarify and identify new immune-related therapeutic targets. High throughput technology has been widely employed to investigate gene expression in numerous tumors, providing a novel method to identify significant genes and explore on tumor progression and initiation.

Here, we report that the expression and prognosis of six key EMT-related genes in cancer, and found high SNAI1 expression was closely related with poorer overall survival in gastrointestinal cancers, and was validated in GEO database. Simultaneously, high expression of SNAI1 correlates with clinical relevance of gastric cancer. Moreover, SNAI1 expression was associated with tumor-infiltrating immune cells in gastrointestinal cancers. In addition, RT-qPCR, immunohistochemistry, and western blot showed SNAI1 expression was higher in gastrointestinal cancers compared to the normal tissues. Finally, high SNAI1 expression was closely related with poorer overall survival and correlates with clinical relevance of gastrointestinal cancers in an independent validation cohort.

E-cad is a Ca^2+^-dependent transmembrane glycoprotein closely related to intercellular adhesion [20]. Studies have found that the expression of E-cad is down-regulated or even completely lost in the malignant progression of epithelial tumors, which leads to the weakening of adhesion between tumor cells and the transformation from benign and non-invasive to malignant and invasive phenotypes [21]. In the study of clinical samples of human gastric cancer, Rosivatz et al. found that the down-regulation of E-cad expression was closely related to the up-regulation of transcription factors Snail, Twist, and SIP1 [22], while Wang et al. also found high expression of transcription factors such as Snail, Twist and Slug in immortalized gastric epithelial cell line Ges-1 and human gastric cancer cell lines MGC-803, BGC-823 and SGC-7901, which were negative for E-cad expression [23]. These transcription factors can bind to the E-box element in the promoter sequence of E-cad gene and inhibit the transcription of E-cad gene. In this study high SNAI1 expression was found in 8 types of cancer (Figure 1B).

Snail, a zinc finger protein, was initially thought to affect EMT, mainly by inhibiting the expression of E-cad, but it was later found that its mechanism of promoting EMT was also related to its down-regulation of epithelial cell characteristic markers (such as claudins, occludins and cytokeratins) and up-regulation of stromal cell characteristic markers (such as fibronectin and vitrinectin) [24]. SNAI1 has a certain value in evaluating the disease progression and survival prognosis of patients with gastric cancer. Its expression level in gastric cancer is significantly correlated with tumor size, degree of differentiation, clinical stage, lymph node and distant metastasis, and the overall survival rate of gastric cancer patients with high expression of SNAI1 is significantly lower than that of patients with low expression of SNAI1 [25]. In cell experiment, it was also found that up-regulation and inhibition of SNAI1 expression could enhance and inhibit the migration and invasion ability of gastric cancer cells in vitro, respectively [26, 27]. In addition, studies have found that cyclooxygenase-2 COX-2) can regulate the expression of E-cad in gastric cancer by affecting nuclear factor κB and SNAI1 pathway, which is also one of the mechanisms of COX-2 regulating the invasion and metastasis of gastric cancer [28]. All these suggest that snail plays an important role in the invasion and metastasis of gastrointestinal cancers.

EMT gives cells the ability to transfer and invade, including stem cell characteristics, reduces apoptosis and aging, and promotes immunosuppression [29]. NF-κB up-regulates the transcription of SNAI1 gene by binding to the promoter region of EMT gene [30]. Related studies have shown that NF-κB can also prevent the apoptosis of epithelial cells with deteriorating tendency in inflammation-related tumors [31]. In addition, the synergistic effect of NF-κB and STAT3 also promotes the generation and metastasis of inflammation-related tumors. STAT3 can not only activate NF-κB, but also induce EMT by regulating SNAI1 to inhibit the activity of E-cadherin promoter [32]. Simultaneously, it is believed that the occurrence of EMT in gastrointestinal cancer cells is the result of the interaction between the acting factors in the tumor microenvironment and gastrointestinal cancer cells. The acting factors transfer the extracellular signal into the cell by binding to the specific receptors on the cell surface, and activate the nuclear transcription factors of SNAI1 through intracellular TGF-β, Wnt, phosphatidylinositol 3-kinase/protein kinase B and other signal transduction pathways. Regulate the expression of downstream genes and the transformation of epithelial cells into stroma, and eventually mediate the transformation of normal epithelial cells into gastrointestinal cancer cells [33].

GI cancers microenvironment plays a critical role in controlling the cancer cell fate, treatment and prognosis. However, the role of EMT in reshaping tumor microenvironment (TME) is unclear. Tumor-associated macrophage is the main type of host immune cells in TME. They regulate tumor colonization and progression by regulating tumor invasion, local tumor immunity and angiogenesis. Hsieh et al. reported that EMT transcription factor Snail directly activates the transcription of miR-21, resulting in miR-21-rich tumor-derived exosome. The exosomes containing miR-21 were phagocytized by CD14+ human monocytes, thus inhibiting the expression of M1 markers and increasing the expression of M2 markers [34]. Faget et al. reported that high expression of SNAI1 reduces T cell homing, changes angiogenesis, leads to hypoxia, and blocks anti-PD1 immunotherapy. Therefore, SNAI1 accelerates the progression of the disease and increases the infiltration of neutrophils in the tumor to maintain the harmful tumor microenvironment [35]. At the same time, CD47 is the direct target of SNAIL1. By using CD47 to block the genetic targeting of SNAI1, the phagocytosis of EMT-activated cells can be rescued [36]. Hence, the important aspect of our study is to emphasize the role of SNAI1 in immune cell infiltration and immune escape in GI cancers. Specifically, SNAI1 expression was negative closely related with infiltrating levels of tumor purity and B cell in STAD, and positively closely related with macrophages in STAD. Moreover, SNAI1 expression was negative closely related with infiltrating levels of tumor purity and B cell in COAD, and positively closely related with CD4+T cells, macrophages, dendritic cells, and neutrophils in COAD. Finally, SNAI1 expression was negative closely related with infiltrating levels of B cell and CD8+ T cells in READ, and positively closely related with CD4+T cells and dendritic cells in READ. Therefore, the cross-talk between SNAI1 and tumor microenvironment might be an important mechanism for the development and progression of GI cancers. Nevertheless, more functional and mechanism experiments are needed for further verification.

In summary, we applied integrated bioinformatics approaches to suggest that high SNAI1 was closely related with prognosis and immune infiltrating levels in gastrointestinal cancer. Therefore, SNAI1 can be used as a prognostic biomarker for determining prognosis and immune infiltration in gastrointestinal cancer, which might provide a novel direction to explore the pathogenesis of gastrointestinal cancer

## MATERIALS AND METHODS

### Data source and processing

Using TIMER (Tumor Immune Estimation Resource, https://cistrome.shinyapps.io/timer/) site to analysis the expression of EMT markers in cancers, the mRNA profiling information is from TCGA (The Cancer Genome Atlas, https://cancergenome.nih.gov/) database [37]. We also Oncomine database (https://www.oncomine.org/resource/login.html) to analysis the expression of SNAI1 in cancers [38]. Bayes test was used to select EMT markers with a change >=2 fold and a *P* value cutoff of 0.05 was defined as statistically significant.

### Survival analysis

GEPIA (Gene Expression Profiling Interactive Analysis, http://gepia.cancer-pku.cn/) site was used to analysis the prognosis of EMT markers in cancers by using the TCGA dataset [39]. A Cox *P*-value < 0.05 was defined as statistically significant. Moreover, we also used Kaplan-Meier plotter database (http://kmplot.com/analysis/index.php?p=service&cancer=gastric) to validate the prognosis of SNAI1 in gastrointestinal cancers [40]. Kaplan-Meier plotter can assess the effect of 54,675 genes on survival using 10,461 cancer samples. These samples include 5,143 breast, 1,816 ovarian, 2,437 lung, and 1,065 gastric cancer samples on the HGU133 Plus 2.0 array with a mean follow-up of 69, 40, 49, and 33 months, respectively. The hazard ratio (HR) with 95% confidence intervals (CI) and log-rank p-value were estimated.

### Immune infiltration

To revealed the immune infiltration of SNAI1 in gastrointestinal cancers, we used TISIDB (tumor-immune system interactions and drugbank, http://cis.hku.hk/TISIDB/index.php) database to infer the relations between abundance of tumor-infiltrating lymphocytes (TILs) and expression of SNAI1. The immune-related signatures of 28 TIL types from Charoentong’s study, which can be viewed in the download page. The relative abundance of TILs was inferred by using genomic variation analysis based on gene expression profiles [41].

### Patients

A total of 440 gastrointestinal cancers patients (200 CRC patients, 240 GC patients) from January 2009 to December 2018, were consecutively recruited in analyses, with clinical information. The clinicopathological characteristics were showed in Table 4.

**Table 4:** Clinicopathologic parameters of patients with CRC and GC in validation cohort.

**Clinicopathologic**	**CRC (n=200)**	**GC (n=240)**
**Gender**		
Male	122	169
Female	78	71
**Age**		
60 years or younger	62	125
Older than 60 years	138	115
**TNM stage**		
I	29	28
II	83	77
III	65	103
IV	23	37
**Pathologic M stage**		
M0	167	203
M1	23	32
**Lymphovascular invasion**		
Negative	163	194
Positive	37	46

### Immunohistochemistry

200 colorectal cancer tissues, 240 gastric cancer tissues and corresponding adjacent tissues were collected to explore the expression of SNAI1 in the tissue samples by using immunohistochemical staining (IHC). IHC staining was performed according to the manufacturer’s instructions. Tissue samples were fixed in 10% formalin embedded in paraffin and cut into slices (4μm). The frozen tissue sections were dewaxed in xylene and rehydrated in descending grades of ethanol. Endogenous peroxidase activity was blocked by 0.3% H_2_O_2_. After heat-induced antigen retrieval, the sections were blocked with 10% rabbit serum for 10min. Subsequently, the sections were incubated with primary antibodies against SNAI1 (1:200) overnight at 4°C, and incubated with an anti-rabbit secondary antibody (1:2,000) for 30 min at 37°C. After that, the sections were visualized with DAB for 10 min at 37°C and counterstained with hematoxylin for 30 sec at 37°C, followed by dehydration with gradient ethanol and sealing with neutral gum.

### Quantitative reverse transcription polymerase chain reaction (qRT-PCR) assays

Total RNA from cells or tissues was isolated using TRIzol (Invitrogen, Canada) reagent, the specific operation is carried out with reference to the instructions for the operation of the kit. RNA (1 μg) was converted into cDNA using the RevertAid First Strand cDNA Synthesis Kit (Takara, China). qRT-PCR was performed using SYBR Green Mixture (Takara, China) in the ABI StepOne-Plus System (ABI7500, USA). Target gene expression was normalized against GAPDH. The primer sequences are 5′-CACCTCCAGACCCACTCAGAT-3′ (sense) and 5′-CCTGAGTGGGGTGGGAGCTTCC-3′ (antisense)

### Western blot

Using the kit to measure protein concentration. An equal amount of protein is added to the gel. The protein was resolved on SDS-PAGE under denatured reducing conditions and transferred to nitrocellulose membranes. The membranes were blocked with 5% non-fat dried milk at room temperature for 30 min and incubated with primary antibodies. The membranes were washed and incubated with horseradish peroxidase-conjugated secondary antibody. Scanning with the Odyssey two-color infrared fluorescence imaging system.

### Statistical analyses

The expression of EMT-related genes in cancer was using TIMER and Oncomine database. The survival curve was generated by GEPIA, PrognoScan, and Kaplan Meier diagrams. One-Way ANOVA was used to compare the expression level of SNAI1 in tumor and normal tissues. To control FDR, the *p*-value was set to less than 0.05. If any independent value is lost, the whole sample is excluded from statistical analysis. Above all, statistical analysis was performed in R version 3.5. p-values < 0.05 were considered statistically significant.

## Supplementary Material

Supplementary Figures

## References

[1] Siegel RL, Miller KD, Jemal A. Cancer statistics, 2020. CA Cancer J Clin. 2020; 70:7–30. 10.3322/caac.21590 31912902

[2] Mehlen P, Puisieux A. Metastasis: a question of life or death. Nat Rev Cancer. 2006; 6:449–58. 10.1038/nrc1886 16723991

[3] Schmidt B, Lee HJ, Ryeom S, Yoon SS. Combining bevacizumab with radiation or chemoradiation for solid tumors: a review of the scientific rationale, and clinical trials. Curr Angiogenes. 2012; 1:169–79. 10.2174/2211552811201030169 24977113 PMC4069862

[4] Miller KD, Nogueira L, Mariotto AB, Rowland JH, Yabroff KR, Alfano CM, Jemal A, Kramer JL, Siegel RL. Cancer treatment and survivorship statistics, 2019. CA Cancer J Clin. 2019; 69:363–85. 10.3322/caac.21565 31184787

[5] Saragoni L, Morgagni P, Gardini A, Marfisi C, Vittimberga G, Garcea D, Scarpi E. Early gastric cancer: diagnosis, staging, and clinical impact. Evaluation of 530 patients. New elements for an updated definition and classification. Gastric Cancer. 2013; 16:549–54. 10.1007/s10120-013-0233-2 23423491

[6] Procaccio L, Schirripa M, Fassan M, Vecchione L, Bergamo F, Prete AA, Intini R, Manai C, Dadduzio V, Boscolo A, Zagonel V, Lonardi S. Immunotherapy in gastrointestinal cancers. Biomed Res Int. 2017; 2017:4346576. 10.1155/2017/4346576 28758114 PMC5512095

[7] Barbee MS, Ogunniyi A, Horvat TZ, Dang TO. Current status and future directions of the immune checkpoint inhibitors ipilimumab, pembrolizumab, and nivolumab in oncology. Ann Pharmacother. 2015; 49:907–37. 10.1177/1060028015586218 25991832

[8] Garon EB, Rizvi NA, Hui R, Leighl N, Balmanoukian AS, Eder JP, Patnaik A, Aggarwal C, Gubens M, Horn L, Carcereny E, Ahn MJ, Felip E, et al, and KEYNOTE-001 Investigators. Pembrolizumab for the treatment of non-small-cell lung cancer. N Engl J Med. 2015; 372:2018–28. 10.1056/NEJMoa1501824 25891174

[9] Rudd CE. A new perspective in cancer immunotherapy: PD-1 on myeloid cells takes center stage in orchestrating immune checkpoint blockade. Sci Immunol. 2020; 5:eaaz8128. 10.1126/sciimmunol.aaz8128 31901075

[10] Grosser R, Cherkassky L, Chintala N, Adusumilli PS. Combination immunotherapy with CAR T cells and checkpoint blockade for the treatment of solid tumors. Cancer Cell. 2019; 36:471–82. 10.1016/j.ccell.2019.09.006 31715131 PMC7171534

[11] Lamouille S, Xu J, Derynck R. Molecular mechanisms of epithelial-mesenchymal transition. Nat Rev Mol Cell Biol. 2014; 15:178–96. 10.1038/nrm3758 24556840 PMC4240281

[12] Zhang Y, Weinberg RA. Epithelial-to-mesenchymal transition in cancer: complexity and opportunities. Front Med. 2018; 12:361–73. 10.1007/s11684-018-0656-6 30043221 PMC6186394

[13] Gonzalez DM, Medici D. Signaling mechanisms of the epithelial-mesenchymal transition. Sci Signal. 2014; 7:re8. 10.1126/scisignal.2005189 25249658 PMC4372086

[14] Xu H, Xu WH, Ren F, Wang J, Wang HK, Cao DL, Shi GH, Qu YY, Zhang HL, Ye DW. Prognostic value of epithelial-mesenchymal transition markers in clear cell renal cell carcinoma. Aging (Albany NY). 2020; 12:866–83. 10.18632/aging.102660 31915310 PMC6977664

[15] Liu Y, Sethi NS, Hinoue T, Schneider BG, Cherniack AD, Sanchez-Vega F, Seoane JA, Farshidfar F, Bowlby R, Islam M, Kim J, Chatila W, Akbani R, et al. Comparative Molecular Analysis of Gastrointestinal Adenocarcinomas. Cancer Cell. 2018; 33:721–735.e8. 10.1016/j.ccell.2018.03.010 29622466 PMC5966039

[16] Azimi F, Scolyer RA, Rumcheva P, Moncrieff M, Murali R, McCarthy SW, Saw RP, Thompson JF. Tumor-infiltrating lymphocyte grade is an independent predictor of sentinel lymph node status and survival in patients with cutaneous melanoma. J Clin Oncol. 2012; 30:2678–83. 10.1200/JCO.2011.37.8539 22711850

[17] Ohtani H. Focus on TILs: prognostic significance of tumor infiltrating lymphocytes in human colorectal cancer. Cancer Immun. 2007; 7:4. 17311363 PMC2935759

[18] Muro K, Chung HC, Shankaran V, Geva R, Catenacci D, Gupta S, Eder JP, Golan T, Le DT, Burtness B, McRee AJ, Lin CC, Pathiraja K, et al. Pembrolizumab for patients with PD-L1-positive advanced gastric cancer (KEYNOTE-012): a multicentre, open-label, phase 1b trial. Lancet Oncol. 2016; 17:717–26. 10.1016/S1470-2045(16)00175-3 27157491

[19] Le DT, Durham JN, Smith KN, Wang H, Bartlett BR, Aulakh LK, Lu S, Kemberling H, Wilt C, Luber BS, Wong F, Azad NS, Rucki AA, et al. Mismatch repair deficiency predicts response of solid tumors to PD-1 blockade. Science. 2017; 357:409–13. 10.1126/science.aan6733 28596308 PMC5576142

[20] Hummel S, Veltman K, Cichon C, Sonnenborn U, Schmidt MA. Differential targeting of the e-cadherin/β-catenin complex by gram-positive probiotic lactobacilli improves epithelial barrier function. Appl Environ Microbiol. 2012; 78:1140–47. 10.1128/AEM.06983-11 22179242 PMC3272997

[21] Berx G, van Roy F. Involvement of members of the cadherin superfamily in cancer. Cold Spring Harb Perspect Biol. 2009; 1:a003129. 10.1101/cshperspect.a003129 20457567 PMC2882122

[22] Rosivatz E, Becker I, Specht K, Fricke E, Luber B, Busch R, Höfler H, Becker KF. Differential expression of the epithelial-mesenchymal transition regulators snail, SIP1, and twist in gastric cancer. Am J Pathol. 2002; 161:1881–91. 10.1016/S0002-9440(10)64464-1 12414534 PMC1850763

[23] Wang BJ, Zhang ZQ, Ke Y. Conversion of cadherin isoforms in cultured human gastric carcinoma cells. World J Gastroenterol. 2006; 12:966–70. 10.3748/wjg.v12.i6.966 16521229 PMC4066166

[24] Zeisberg M, Neilson EG. Biomarkers for epithelial-mesenchymal transitions. J Clin Invest. 2009; 119:1429–37. 10.1172/JCI36183 19487819 PMC2689132

[25] He H, Chen W, Wang X, Wang C, Liu F, Shen Z, Xu J, Gu J, Sun Y. Snail is an independent prognostic predictor for progression and patient survival of gastric cancer. Cancer Sci. 2012; 103:1296–303. 10.1111/j.1349-7006.2012.02295.x 22471696 PMC7659386

[26] Shin NR, Jeong EH, Choi CI, Moon HJ, Kwon CH, Chu IS, Kim GH, Jeon TY, Kim DH, Lee JH, Park DY. Overexpression of snail is associated with lymph node metastasis and poor prognosis in patients with gastric cancer. BMC Cancer. 2012; 12:521. 10.1186/1471-2407-12-521 23151184 PMC3552976

[27] Guo HM, Zhang XQ, Xu CH, Zou XP. Inhibition of invasion and metastasis of gastric cancer cells through snail targeting artificial microRNA interference. Asian Pac J Cancer Prev. 2011; 12:3433–38. 22471493

[28] Chen Z, Liu M, Liu X, Huang S, Li L, Song B, Li H, Ren Q, Hu Z, Zhou Y, Qiao L. COX-2 regulates e-cadherin expression through the NF-κB/snail signaling pathway in gastric cancer. Int J Mol Med. 2013; 32:93–100. 10.3892/ijmm.2013.1376 23670240

[29] Dongre A, Rashidian M, Reinhardt F, Bagnato A, Keckesova Z, Ploegh HL, Weinberg RA. Epithelial-to-mesenchymal transition contributes to immunosuppression in breast carcinomas. Cancer Res. 2017; 77:3982–89. 10.1158/0008-5472.CAN-16-3292 28428275 PMC5541771

[30] Liu J, Wu Z, Han D, Wei C, Liang Y, Jiang T, Chen L, Sha M, Cao Y, Huang F, Geng X, Yu J, Shen Y, et al. Mesencephalic Astrocyte-Derived Neurotrophic Factor Inhibits Liver Cancer Through Small Ubiquitin-Related Modifier (SUMO)ylation-Related Suppression of NF-κB/Snail Signaling Pathway and Epithelial-Mesenchymal Transition. Hepatology. 2020; 71:1262–1278. 10.1002/hep.30917 31469428 PMC7187412

[31] Greten FR, Eckmann L, Greten TF, Park JM, Li ZW, Egan LJ, Kagnoff MF, Karin M. IKKbeta links inflammation and tumorigenesis in a mouse model of colitis-associated cancer. Cell. 2004; 118:285–96. 10.1016/j.cell.2004.07.013 15294155

[32] Xiong H, Hong J, Du W, Lin YW, Ren LL, Wang YC, Su WY, Wang JL, Cui Y, Wang ZH, Fang JY. Roles of STAT3 and ZEB1 proteins in e-cadherin down-regulation and human colorectal cancer epithelial-mesenchymal transition. J Biol Chem. 2012; 287:5819–32. 10.1074/jbc.M111.295964 22205702 PMC3285352

[33] Bates RC, Mercurio AM. Tumor necrosis factor-alpha stimulates the epithelial-to-mesenchymal transition of human colonic organoids. Mol Biol Cell. 2003; 14:1790–800. 10.1091/mbc.e02-09-0583 12802055 PMC165077

[34] Hsieh CH, Tai SK, Yang MH. Snail-overexpressing cancer cells promote M2-like polarization of tumor-associated macrophages by delivering MiR-21-abundant exosomes. Neoplasia. 2018; 20:775–88. 10.1016/j.neo.2018.06.004 29981499 PMC6031090

[35] Faget J, Groeneveld S, Boivin G, Sankar M, Zangger N, Garcia M, Guex N, Zlobec I, Steiner L, Piersigilli A, Xenarios I, Meylan E. Neutrophils and snail orchestrate the establishment of a pro-tumor microenvironment in lung cancer. Cell Rep. 2017; 21:3190–204. 10.1016/j.celrep.2017.11.052 29241546

[36] Noman MZ, Van Moer K, Marani V, Gemmill RM, Tranchevent LC, Azuaje F, Muller A, Chouaib S, Thiery JP, Berchem G, Janji B. CD47 is a direct target of SNAI1 and ZEB1 and its blockade activates the phagocytosis of breast cancer cells undergoing EMT. Oncoimmunology. 2018; 7:e1345415. 10.1080/2162402X.2017.1345415 29632713 PMC5889210

[37] Li T, Fan J, Wang B, Traugh N, Chen Q, Liu JS, Li B, Liu XS. TIMER: a web server for comprehensive analysis of tumor-infiltrating immune cells. Cancer Res. 2017; 77:e108–10. 10.1158/0008-5472.CAN-17-0307 29092952 PMC6042652

[38] Rhodes DR, Kalyana-Sundaram S, Mahavisno V, Varambally R, Yu J, Briggs BB, Barrette TR, Anstet MJ, Kincead-Beal C, Kulkarni P, Varambally S, Ghosh D, Chinnaiyan AM. Oncomine 3.0: genes, pathways, and networks in a collection of 18,000 cancer gene expression profiles. Neoplasia. 2007; 9:166–80. 10.1593/neo.07112 17356713 PMC1813932

[39] Tang Z, Li C, Kang B, Gao G, Li C, Zhang Z. GEPIA: a web server for cancer and normal gene expression profiling and interactive analyses. Nucleic Acids Res. 2017; 45:W98–102. 10.1093/nar/gkx247 28407145 PMC5570223

[40] Szász AM, Lánczky A, Nagy Á, Förster S, Hark K, Green JE, Boussioutas A, Busuttil R, Szabó A, Győrffy B. Cross-validation of survival associated biomarkers in gastric cancer using transcriptomic data of 1,065 patients. Oncotarget. 2016; 7:49322–33. 10.18632/oncotarget.10337 27384994 PMC5226511

[41] Ru B, Wong CN, Tong Y, Zhong JY, Zhong SS, Wu WC, Chu KC, Wong CY, Lau CY, Chen I, Chan NW, Zhang J. TISIDB: an integrated repository portal for tumor-immune system interactions. Bioinformatics. 2019; 35:4200–02. 10.1093/bioinformatics/btz210 30903160

