# Supplementary material for: SNAI1 is a prognostic biomarker and correlated with immune infiltrates in gastrointestinal cancers
Source: Aging (Albany NY). 2020 Aug 21;12(17):17167–208. doi: 10.18632/aging.103667 (PMC7521489; doi:10.18632/aging.103667)
Supplement: Supplementary Figures [file aging-12-103667-s001..pdf]

SUPPLEMENTARY FIGURES

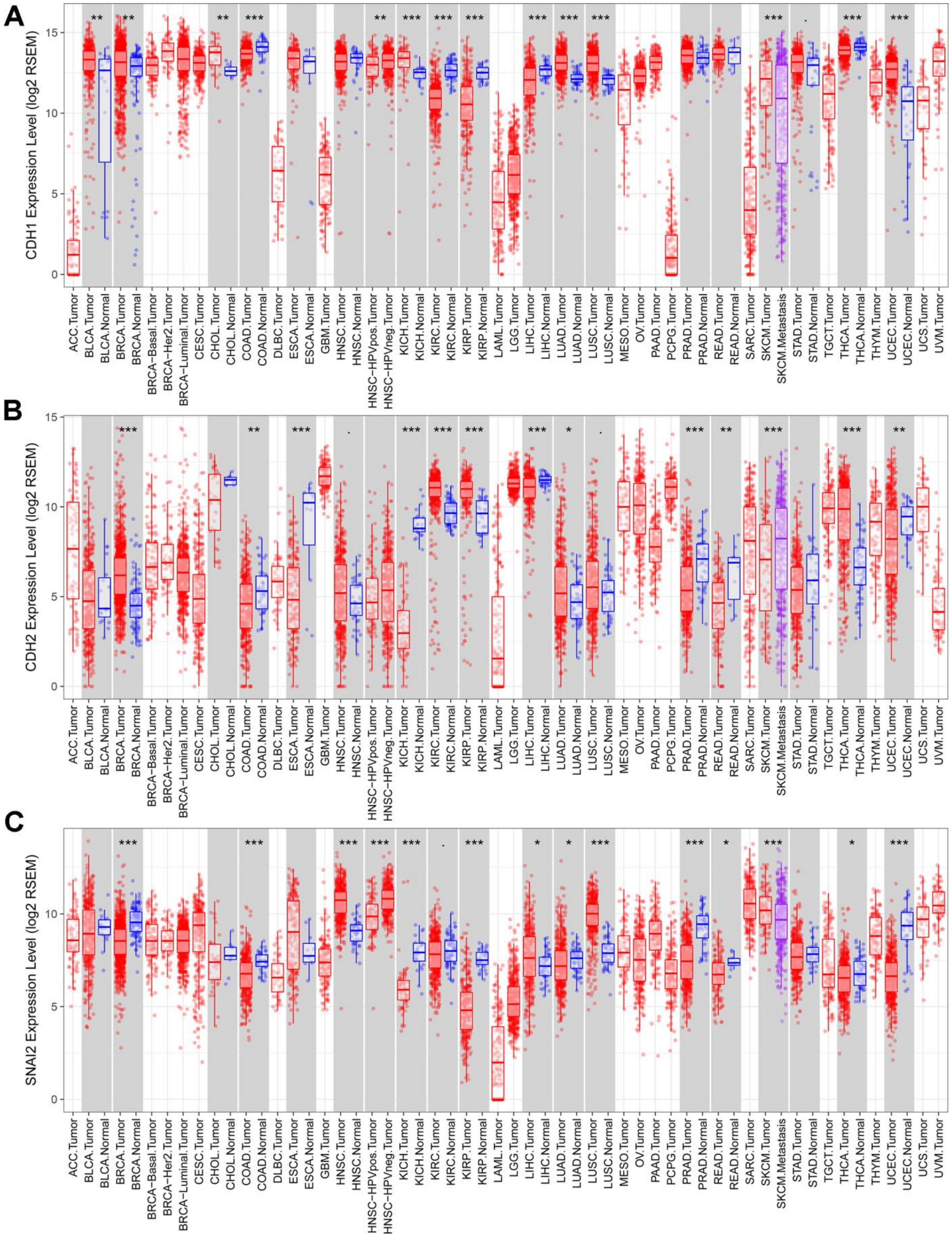

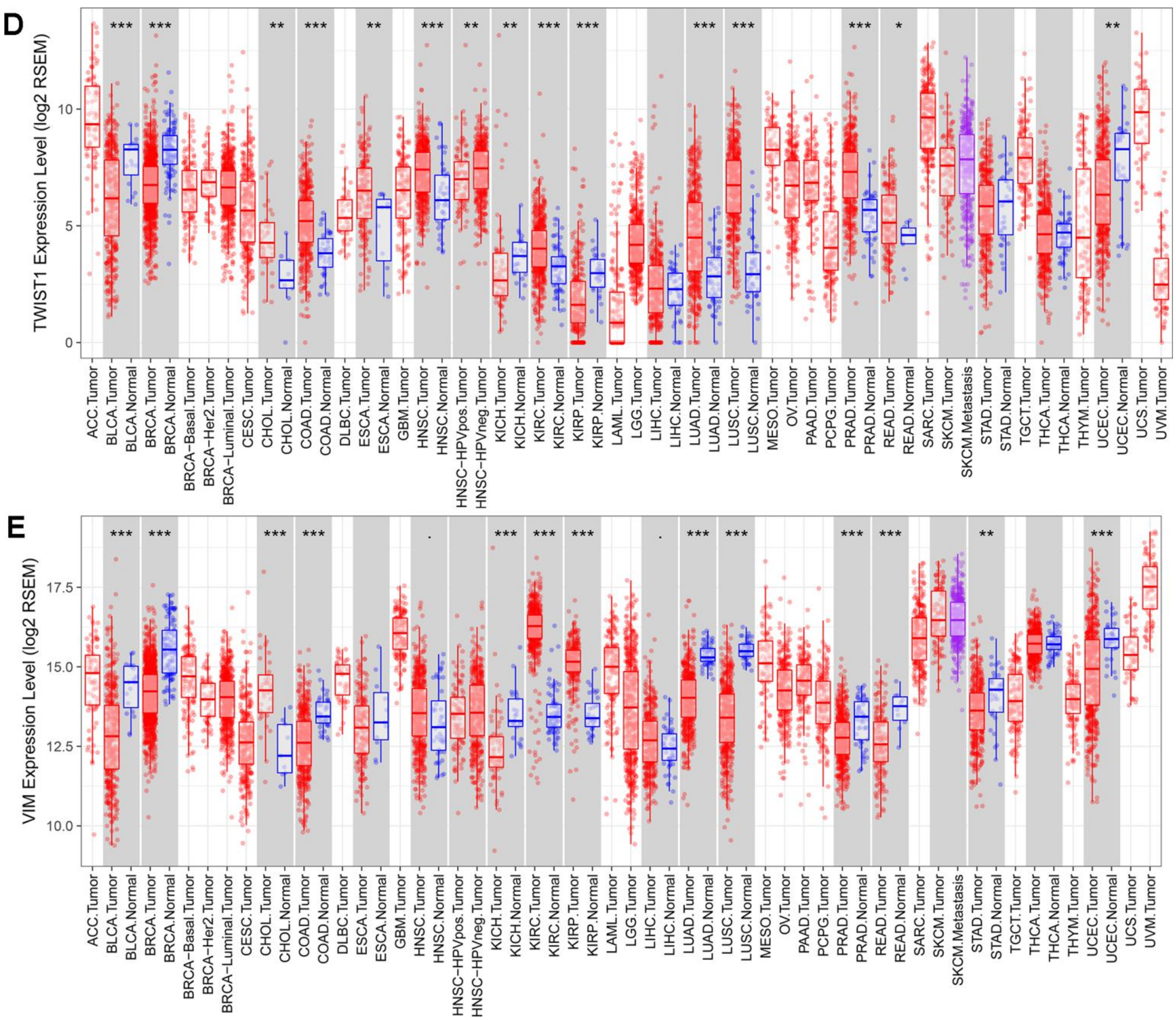

Supplementary Figure 1. The five EMT markers expression levels in different types of human cancers in TCGA dataset.

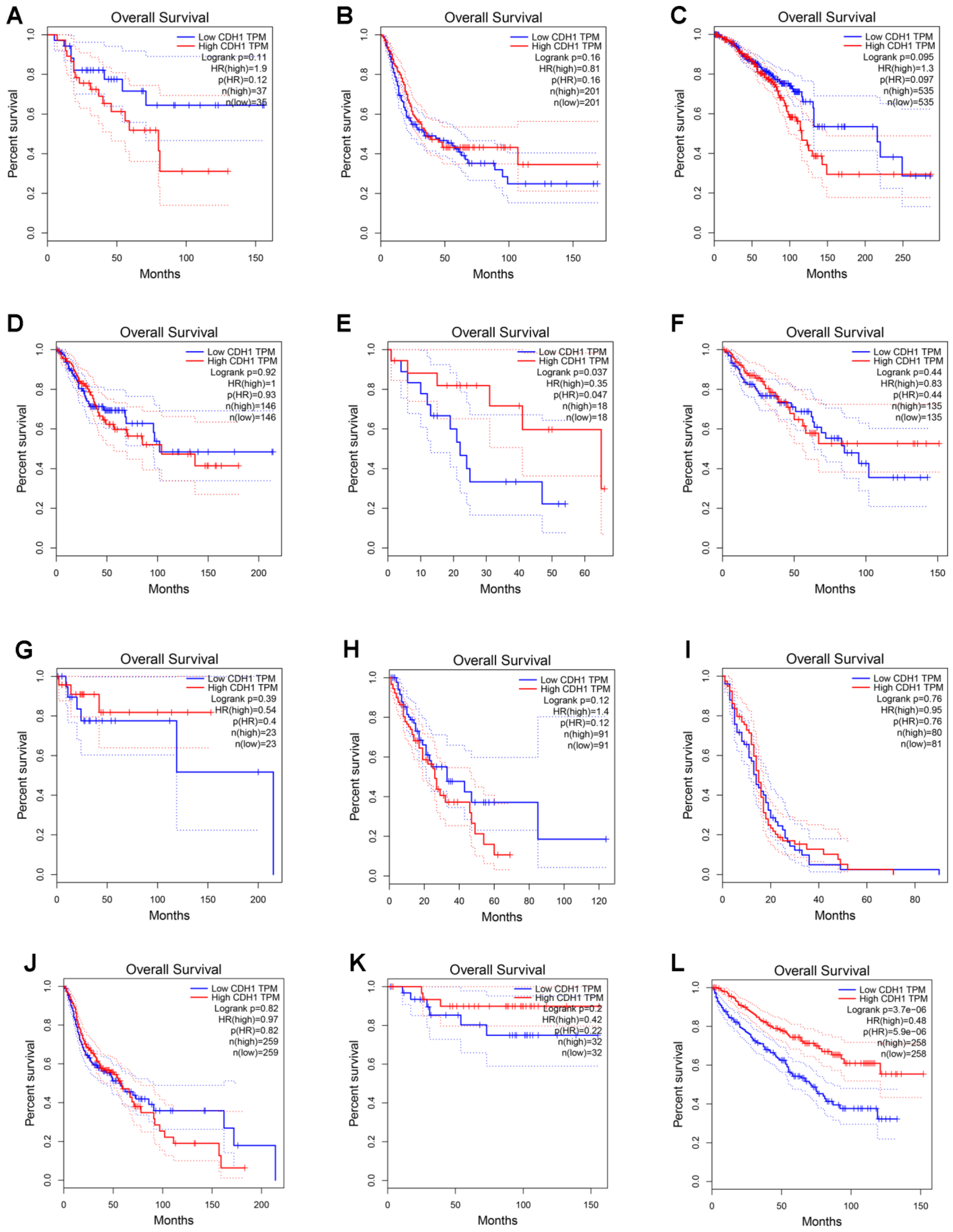
A
Overall Survival
Low CDH1 TPM
High CDH1 TPM
Logrank p=0.11
HR(high)=1.9
p(HR)=0.12
n(high)=37
n(low)=36
Percent survival
Months
B
Overall Survival
Low CDH1 TPM
High CDH1 TPM
Logrank p=0.16
HR(high)=0.81
p(HR)=0.16
n(high)=201
n(low)=201
C
Overall Survival
Low CDH1 TPM
High CDH1 TPM
Logrank p=0.095
HR(high)=1.3
p(HR)=0.097
n(high)=535
n(low)=535
D
Overall Survival
Low CDH1 TPM
High CDH1 TPM
Logrank p=0.92
HR(high)=1
p(HR)=0.93
n(high)=146
n(low)=146
E
Overall Survival
Low CDH1 TPM
High CDH1 TPM
Logrank p=0.037
HR(high)=0.35
p(HR)=0.047
n(high)=18
n(low)=18
F
Overall Survival
Low CDH1 TPM
High CDH1 TPM
Logrank p=0.44
HR(high)=0.83
p(HR)=0.44
n(high)=135
n(low)=135
G
Overall Survival
Low CDH1 TPM
High CDH1 TPM
Logrank p=0.39
HR(high)=0.54
p(HR)=0.4
n(high)=23
n(low)=23
H
Overall Survival
Low CDH1 TPM
High CDH1 TPM
Logrank p=0.12
HR(high)=1.4
p(HR)=0.12
n(high)=91
n(low)=91
I
Overall Survival
Low CDH1 TPM
High CDH1 TPM
Logrank p=0.76
HR(high)=0.95
p(HR)=0.76
n(high)=80
n(low)=81
J
Overall Survival
Low CDH1 TPM
High CDH1 TPM
Logrank p=0.82
HR(high)=0.97
p(HR)=0.82
n(high)=259
n(low)=259
K
Overall Survival
Low CDH1 TPM
High CDH1 TPM
Logrank p=0.2
HR(high)=0.42
p(HR)=0.22
n(high)=32
n(low)=32
L
Overall Survival
Low CDH1 TPM
High CDH1 TPM
Logrank p=3.7e−06
HR(high)=0.48
p(HR)=5.9e−06
n(high)=258
n(low)=258

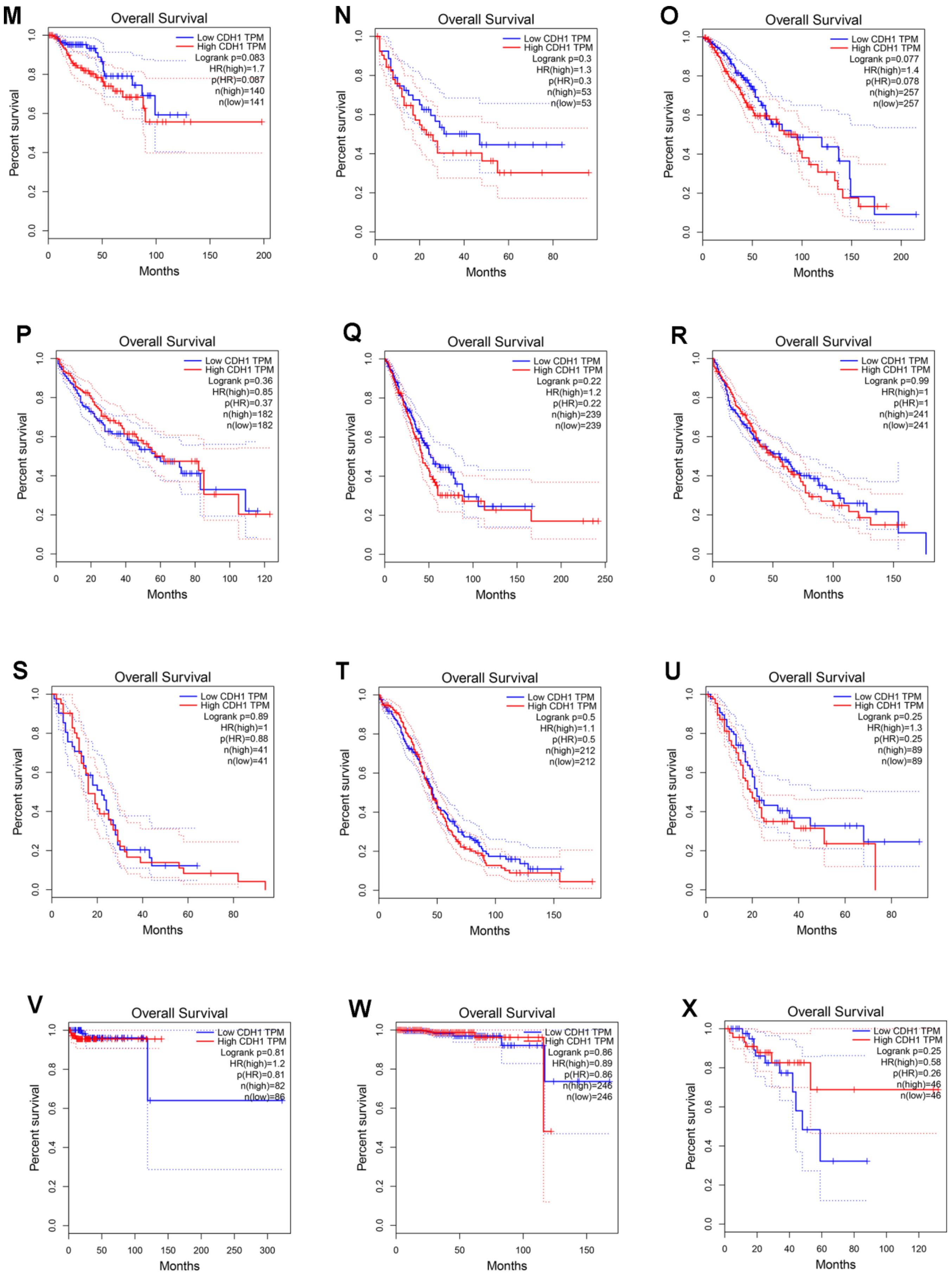
M
Overall Survival
Low CDH1 TPM
High CDH1 TPM
Logrank p=0.083
HR(high)=1.7
p(HR)=0.087
n(high)=140
n(low)=141
N
Overall Survival
Low CDH1 TPM
High CDH1 TPM
Logrank p=0.3
HR(high)=1.3
p(HR)=0.3
n(high)=53
n(low)=53
O
Overall Survival
Low CDH1 TPM
High CDH1 TPM
Logrank p=0.077
HR(high)=1.4
p(HR)=0.078
n(high)=257
n(low)=257
P
Overall Survival
Low CDH1 TPM
High CDH1 TPM
Logrank p=0.36
HR(high)=0.85
p(HR)=0.37
n(high)=182
n(low)=182
Q
Overall Survival
Low CDH1 TPM
High CDH1 TPM
Logrank p=0.22
HR(high)=1.2
p(HR)=0.22
n(high)=239
n(low)=239
R
Overall Survival
Low CDH1 TPM
High CDH1 TPM
Logrank p=0.99
HR(high)=1
p(HR)=1
n(high)=241
n(low)=241
S
Overall Survival
Low CDH1 TPM
High CDH1 TPM
Logrank p=0.89
HR(high)=1
p(HR)=0.88
n(high)=41
n(low)=41
T
Overall Survival
Low CDH1 TPM
High CDH1 TPM
Logrank p=0.5
HR(high)=1.1
p(HR)=0.5
n(high)=212
n(low)=212
U
Overall Survival
Low CDH1 TPM
High CDH1 TPM
Logrank p=0.25
HR(high)=1.3
p(HR)=0.25
n(high)=89
n(low)=89
V
Overall Survival
Low CDH1 TPM
High CDH1 TPM
Logrank p=0.81
HR(high)=1.2
p(HR)=0.81
n(high)=82
n(low)=86
W
Overall Survival
Low CDH1 TPM
High CDH1 TPM
Logrank p=0.86
HR(high)=0.89
p(HR)=0.86
n(high)=246
n(low)=246
X
Overall Survival
Low CDH1 TPM
High CDH1 TPM
Logrank p=0.25
HR(high)=0.58
p(HR)=0.26
n(high)=46
n(low)=46
Percent survival
Months

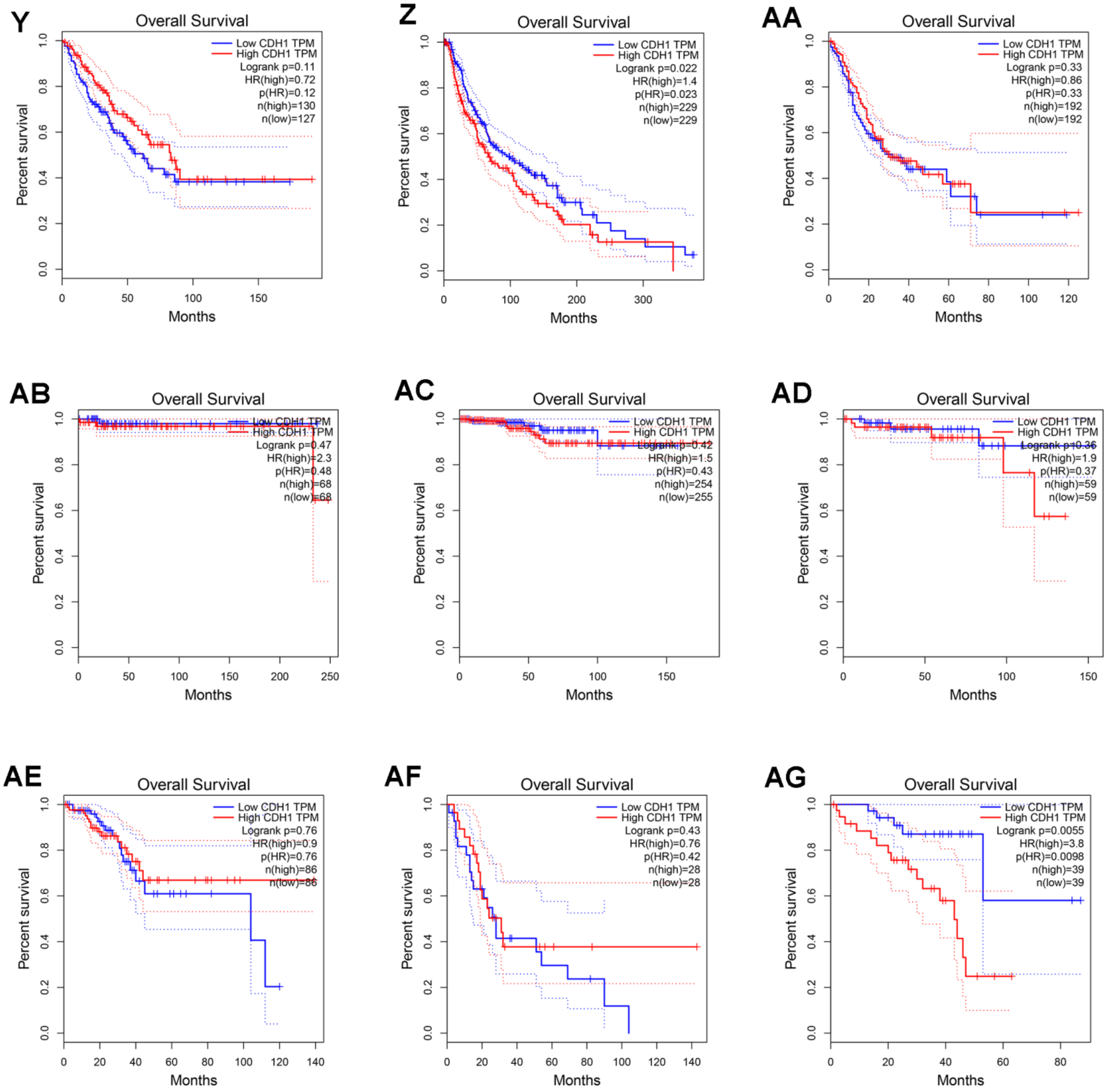

Supplementary Figure 2. Kaplan-Meier survival curves comparing the high and low expression of CDH1 in different types of human cancers in TCGA dataset.

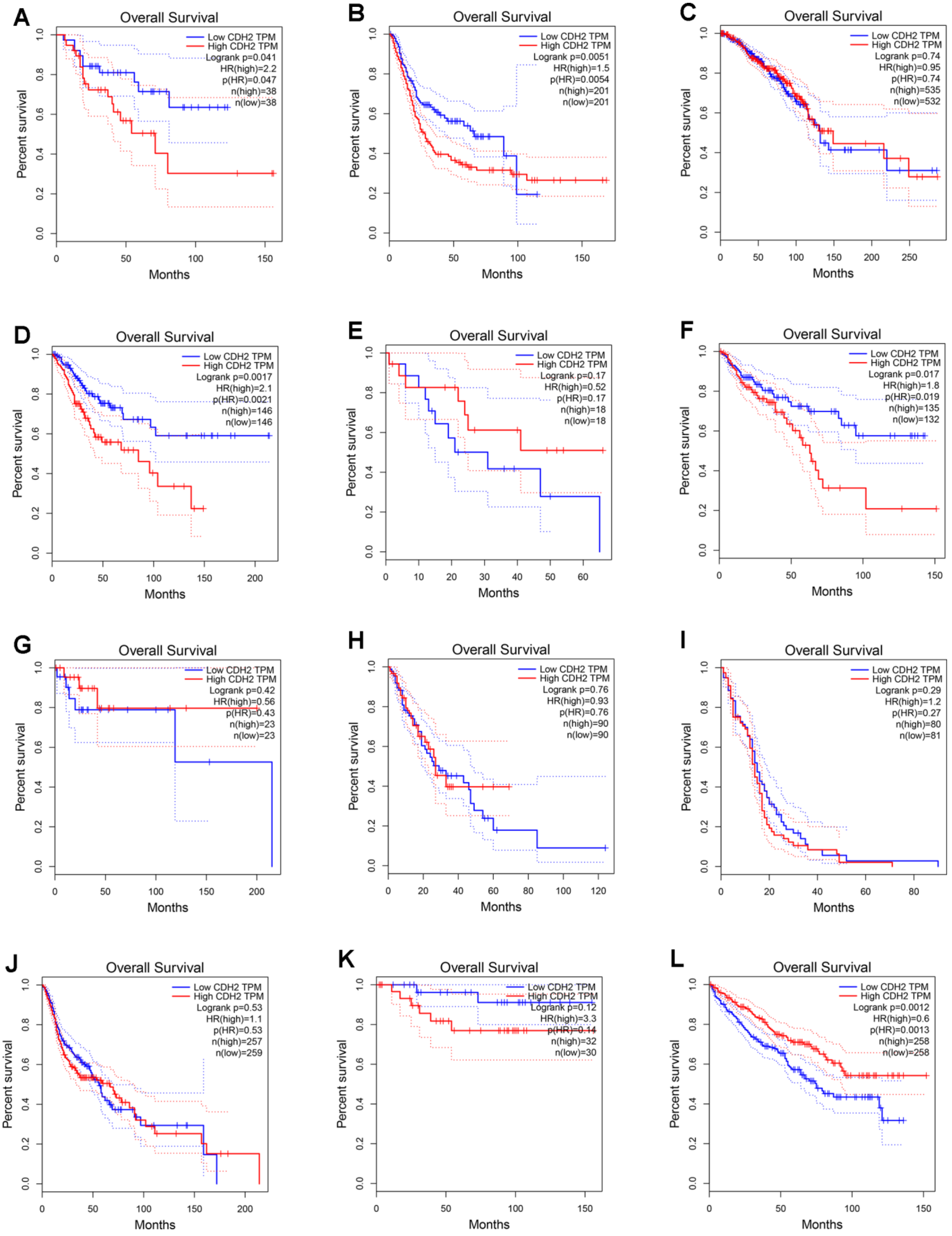
A
Overall Survival
Low CDH2 TPM
High CDH2 TPM
Logrank p=0.041
HR(high)=2.2
p(HR)=0.047
n(high)=38
n(low)=38
Percent survival
Months
B
Overall Survival
Low CDH2 TPM
High CDH2 TPM
Logrank p=0.0051
HR(high)=1.5
p(HR)=0.0054
n(high)=201
n(low)=201
Percent survival
Months
C
Overall Survival
Low CDH2 TPM
High CDH2 TPM
Logrank p=0.74
HR(high)=0.95
p(HR)=0.74
n(high)=535
n(low)=532
Percent survival
Months
D
Overall Survival
Low CDH2 TPM
High CDH2 TPM
Logrank p=0.0017
HR(high)=2.1
p(HR)=0.0021
n(high)=146
n(low)=146
Percent survival
Months
E
Overall Survival
Low CDH2 TPM
High CDH2 TPM
Logrank p=0.17
HR(high)=0.52
p(HR)=0.17
n(high)=18
n(low)=18
Percent survival
Months
F
Overall Survival
Low CDH2 TPM
High CDH2 TPM
Logrank p=0.017
HR(high)=1.8
p(HR)=0.019
n(high)=135
n(low)=132
Percent survival
Months
G
Overall Survival
Low CDH2 TPM
High CDH2 TPM
Logrank p=0.42
HR(high)=0.56
p(HR)=0.43
n(high)=23
n(low)=23
Percent survival
Months
H
Overall Survival
Low CDH2 TPM
High CDH2 TPM
Logrank p=0.76
HR(high)=0.93
p(HR)=0.76
n(high)=90
n(low)=90
Percent survival
Months
I
Overall Survival
Low CDH2 TPM
High CDH2 TPM
Logrank p=0.29
HR(high)=1.2
p(HR)=0.27
n(high)=80
n(low)=81
Percent survival
Months
J
Overall Survival
Low CDH2 TPM
High CDH2 TPM
Logrank p=0.53
HR(high)=1.1
p(HR)=0.53
n(high)=257
n(low)=259
Percent survival
Months
K
Overall Survival
Low CDH2 TPM
High CDH2 TPM
Logrank p=0.12
HR(high)=3.3
p(HR)=0.14
n(high)=32
n(low)=30
Percent survival
Months
L
Overall Survival
Low CDH2 TPM
High CDH2 TPM
Logrank p=0.0012
HR(high)=0.6
p(HR)=0.0013
n(high)=258
n(low)=258
Percent survival
Months

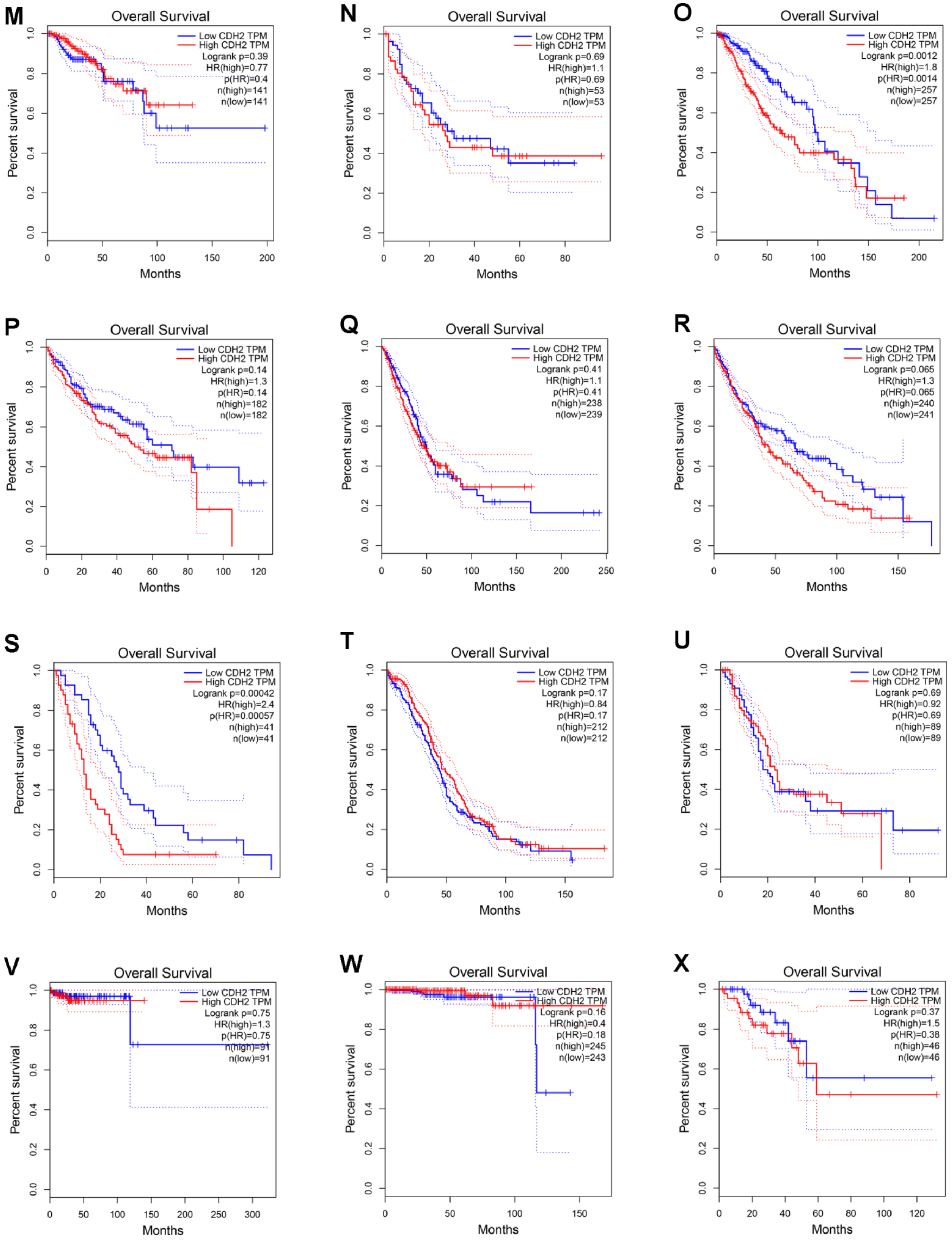
M
Overall Survival
Low CDH2 TPM
High CDH2 TPM
Logrank p=0.39
HR(high)=0.77
p(HR)=0.4
n(high)=141
n(low)=141
Percent survival
Months
N
Overall Survival
Low CDH2 TPM
High CDH2 TPM
Logrank p=0.69
HR(high)=1.1
p(HR)=0.69
n(high)=53
n(low)=53
Percent survival
Months
O
Overall Survival
Low CDH2 TPM
High CDH2 TPM
Logrank p=0.0012
HR(high)=1.8
p(HR)=0.0014
n(high)=257
n(low)=257
Percent survival
Months
P
Overall Survival
Low CDH2 TPM
High CDH2 TPM
Logrank p=0.14
HR(high)=1.3
p(HR)=0.14
n(high)=182
n(low)=182
Percent survival
Months
Q
Overall Survival
Low CDH2 TPM
High CDH2 TPM
Logrank p=0.41
HR(high)=1.1
p(HR)=0.41
n(high)=238
n(low)=239
Percent survival
Months
R
Overall Survival
Low CDH2 TPM
High CDH2 TPM
Logrank p=0.065
HR(high)=1.3
p(HR)=0.065
n(high)=240
n(low)=241
Percent survival
Months
S
Overall Survival
Low CDH2 TPM
High CDH2 TPM
Logrank p=0.00042
HR(high)=2.4
p(HR)=0.00057
n(high)=41
n(low)=41
Percent survival
Months
T
Overall Survival
Low CDH2 TPM
High CDH2 TPM
Logrank p=0.17
HR(high)=0.84
p(HR)=0.17
n(high)=212
n(low)=212
Percent survival
Months
U
Overall Survival
Low CDH2 TPM
High CDH2 TPM
Logrank p=0.69
HR(high)=0.92
p(HR)=0.69
n(high)=89
n(low)=89
Percent survival
Months
V
Overall Survival
Low CDH2 TPM
High CDH2 TPM
Logrank p=0.75
HR(high)=1.3
p(HR)=0.75
n(high)=91
n(low)=91
Percent survival
Months
W
Overall Survival
Low CDH2 TPM
High CDH2 TPM
Logrank p=0.16
HR(high)=0.4
p(HR)=0.18
n(high)=245
n(low)=243
Percent survival
Months
X
Overall Survival
Low CDH2 TPM
High CDH2 TPM
Logrank p=0.37
HR(high)=1.5
p(HR)=0.38
n(high)=46
n(low)=46
Percent survival
Months

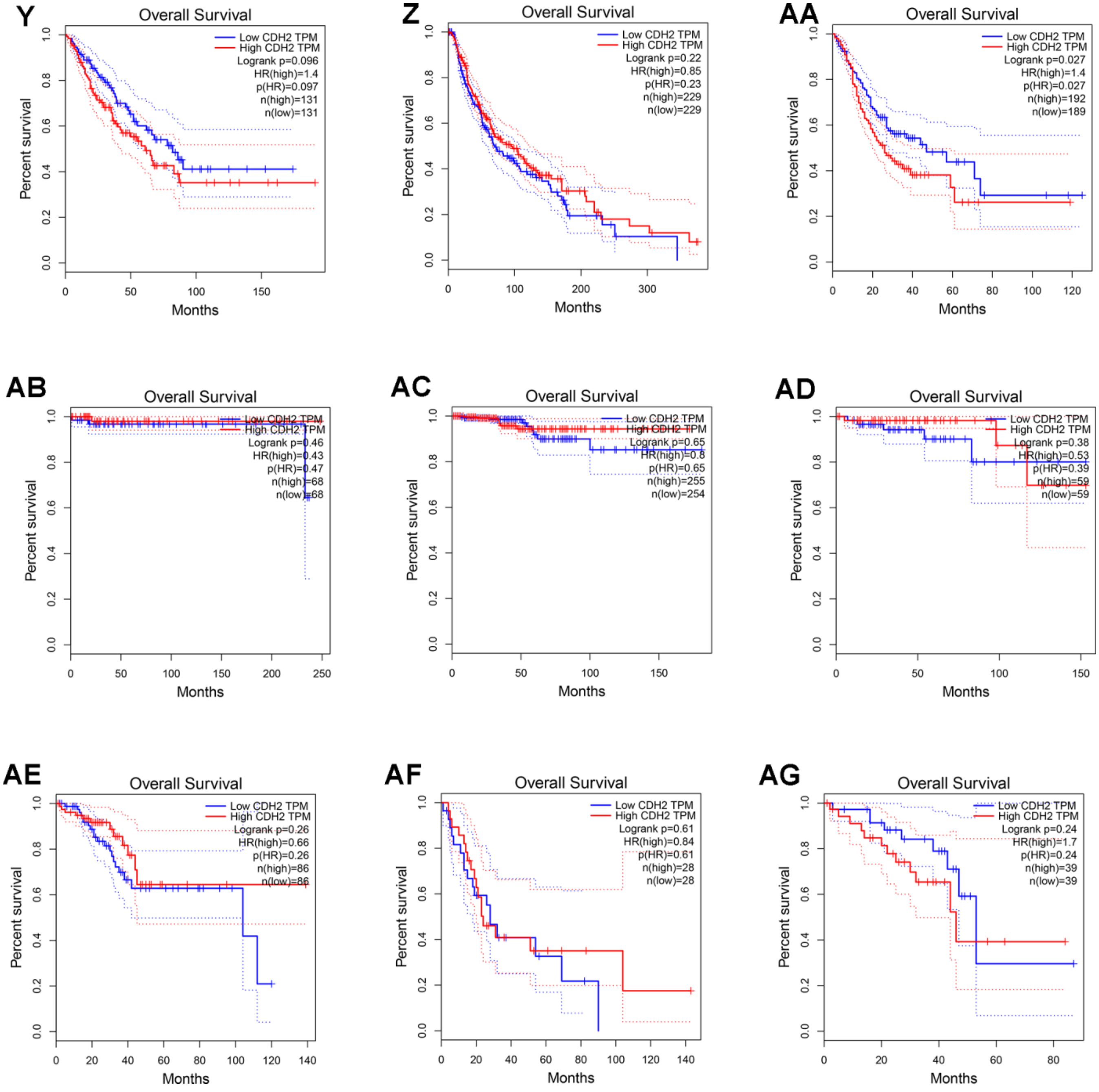

Supplementary Figure 3. Kaplan-Meier survival curves comparing the high and low expression of CDH2 in different types of human cancers in TCGA dataset.

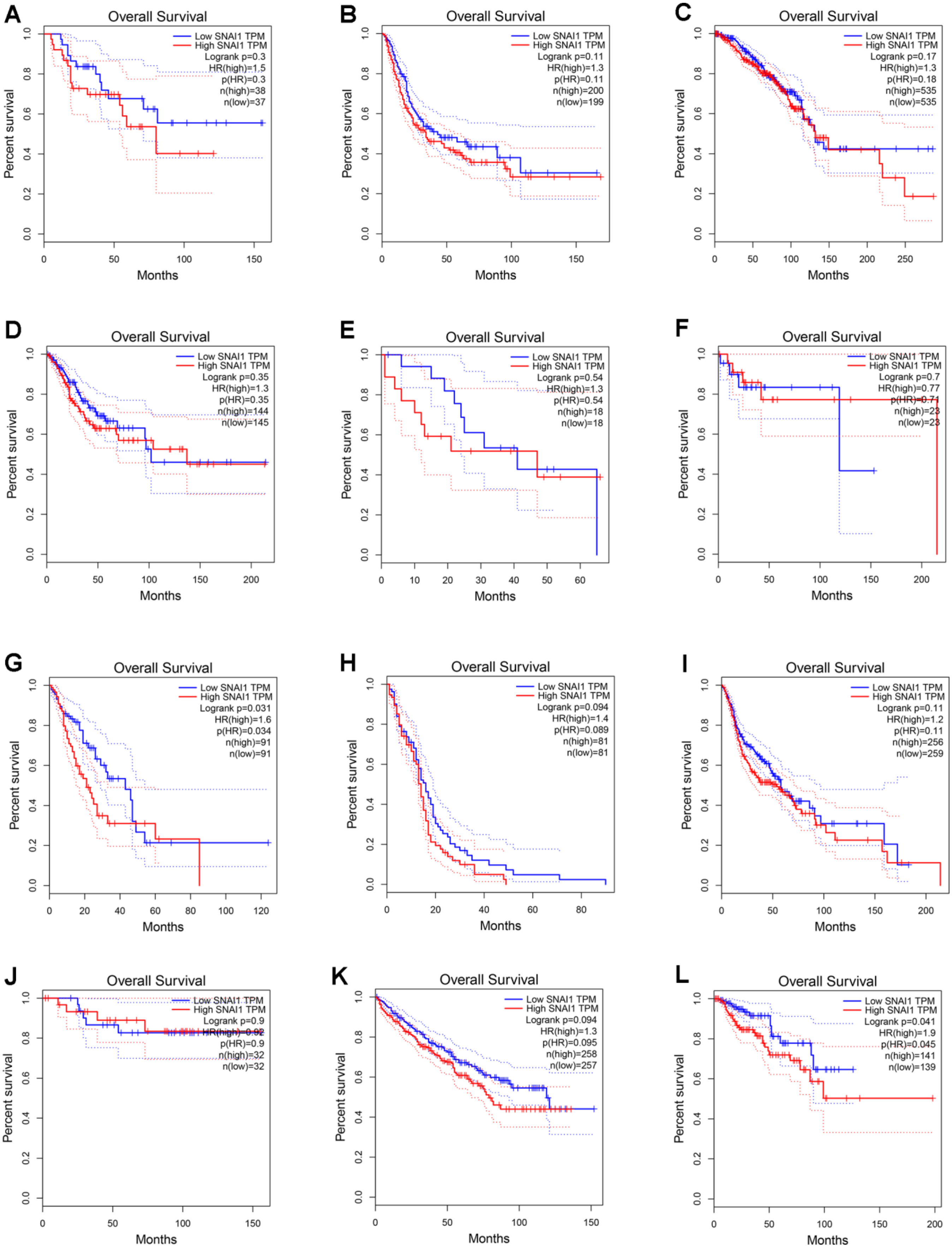
A
Overall Survival
Low SNAI1 TPM
High SNAI1 TPM
Logrank p=0.3
HR(high)=1.5
p(HR)=0.3
n(high)=38
n(low)=37
Percent survival
Months
B
Overall Survival
Low SNAI1 TPM
High SNAI1 TPM
Logrank p=0.11
HR(high)=1.3
p(HR)=0.11
n(high)=200
n(low)=199
Percent survival
Months
C
Overall Survival
Low SNAI1 TPM
High SNAI1 TPM
Logrank p=0.17
HR(high)=1.3
p(HR)=0.18
n(high)=535
n(low)=535
Percent survival
Months
D
Overall Survival
Low SNAI1 TPM
High SNAI1 TPM
Logrank p=0.35
HR(high)=1.3
p(HR)=0.35
n(high)=144
n(low)=145
Percent survival
Months
E
Overall Survival
Low SNAI1 TPM
High SNAI1 TPM
Logrank p=0.54
HR(high)=1.3
p(HR)=0.54
n(high)=18
n(low)=18
Percent survival
Months
F
Overall Survival
Low SNAI1 TPM
High SNAI1 TPM
Logrank p=0.7
HR(high)=0.77
p(HR)=0.71
n(high)=23
n(low)=23
Percent survival
Months
G
Overall Survival
Low SNAI1 TPM
High SNAI1 TPM
Logrank p=0.031
HR(high)=1.6
p(HR)=0.034
n(high)=91
n(low)=91
Percent survival
Months
H
Overall Survival
Low SNAI1 TPM
High SNAI1 TPM
Logrank p=0.094
HR(high)=1.4
p(HR)=0.089
n(high)=81
n(low)=81
Percent survival
Months
I
Overall Survival
Low SNAI1 TPM
High SNAI1 TPM
Logrank p=0.11
HR(high)=1.2
p(HR)=0.11
n(high)=256
n(low)=259
Percent survival
Months
J
Overall Survival
Low SNAI1 TPM
High SNAI1 TPM
Logrank p=0.9
HR(high)=0.92
p(HR)=0.9
n(high)=32
n(low)=32
Percent survival
Months
K
Overall Survival
Low SNAI1 TPM
High SNAI1 TPM
Logrank p=0.094
HR(high)=1.3
p(HR)=0.095
n(high)=258
n(low)=257
Percent survival
Months
L
Overall Survival
Low SNAI1 TPM
High SNAI1 TPM
Logrank p=0.041
HR(high)=1.9
p(HR)=0.045
n(high)=141
n(low)=139
Percent survival
Months

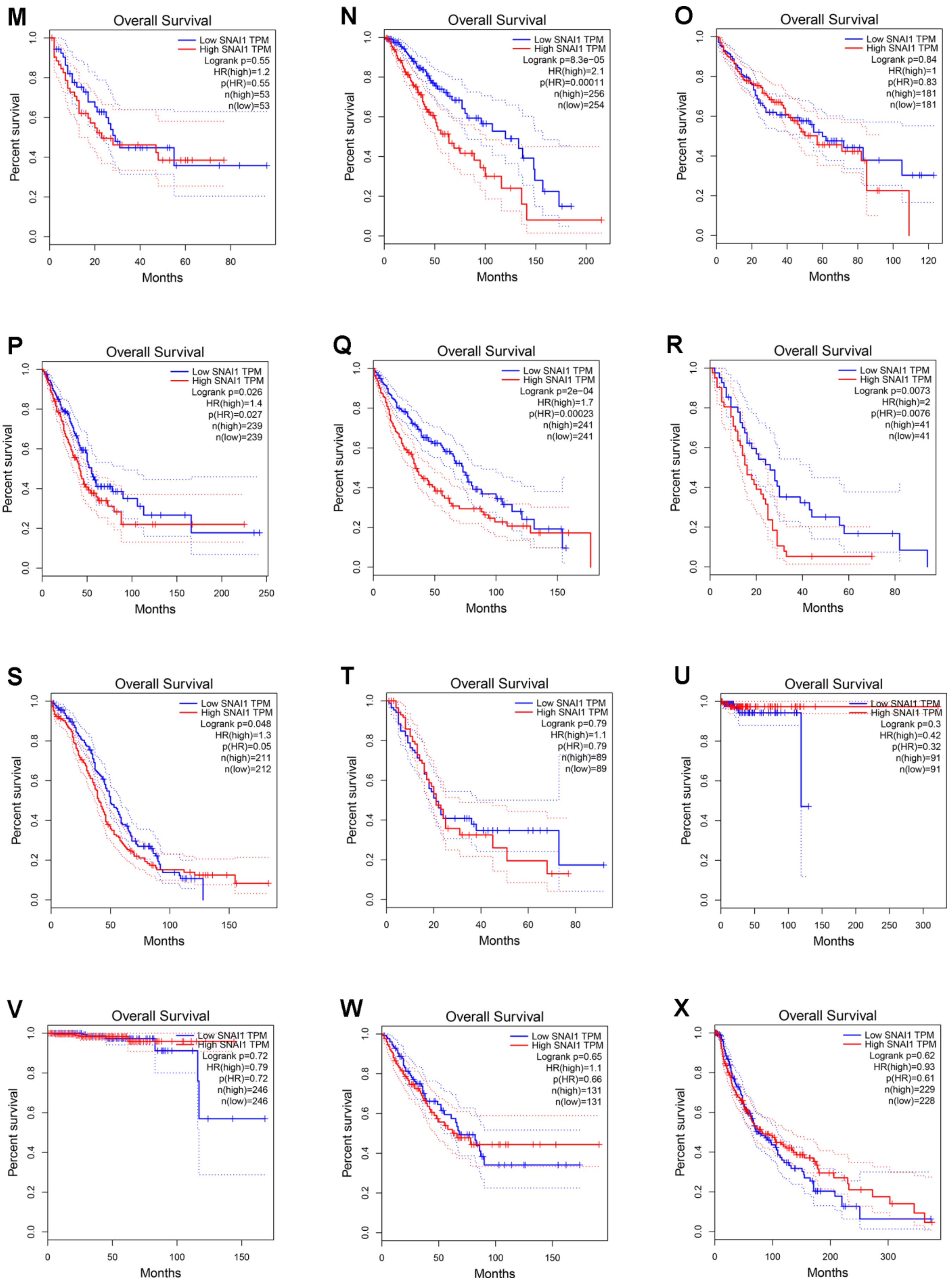

M
Overall Survival
Low SNAI1 TPM
High SNAI1 TPM
Logrank p=0.55
HR(high)=1.2
p(HR)=0.55
n(high)=53
n(low)=53
Percent survival
Months
N
Overall Survival
Low SNAI1 TPM
High SNAI1 TPM
Logrank p=8.3e−05
HR(high)=2.1
p(HR)=0.00011
n(high)=256
n(low)=254
Percent survival
Months
O
Overall Survival
Low SNAI1 TPM
High SNAI1 TPM
Logrank p=0.84
HR(high)=1
p(HR)=0.83
n(high)=181
n(low)=181
Percent survival
Months
P
Overall Survival
Low SNAI1 TPM
High SNAI1 TPM
Logrank p=0.026
HR(high)=1.4
p(HR)=0.027
n(high)=239
n(low)=239
Percent survival
Months
Q
Overall Survival
Low SNAI1 TPM
High SNAI1 TPM
Logrank p=2e−04
HR(high)=1.7
p(HR)=0.00023
n(high)=241
n(low)=241
Percent survival
Months
R
Overall Survival
Low SNAI1 TPM
High SNAI1 TPM
Logrank p=0.0073
HR(high)=2
p(HR)=0.0076
n(high)=41
n(low)=41
Percent survival
Months
S
Overall Survival
Low SNAI1 TPM
High SNAI1 TPM
Logrank p=0.048
HR(high)=1.3
p(HR)=0.05
n(high)=211
n(low)=212
Percent survival
Months
T
Overall Survival
Low SNAI1 TPM
High SNAI1 TPM
Logrank p=0.79
HR(high)=1.1
p(HR)=0.79
n(high)=89
n(low)=89
Percent survival
Months
U
Overall Survival
Low SNAI1 TPM
High SNAI1 TPM
Logrank p=0.3
HR(high)=0.42
p(HR)=0.32
n(high)=91
n(low)=91
Percent survival
Months
V
Overall Survival
Low SNAI1 TPM
High SNAI1 TPM
Logrank p=0.72
HR(high)=0.79
p(HR)=0.72
n(high)=246
n(low)=246
Percent survival
Months
W
Overall Survival
Low SNAI1 TPM
High SNAI1 TPM
Logrank p=0.65
HR(high)=1.1
p(HR)=0.66
n(high)=131
n(low)=131
Percent survival
Months
X
Overall Survival
Low SNAI1 TPM
High SNAI1 TPM
Logrank p=0.62
HR(high)=0.93
p(HR)=0.61
n(high)=229
n(low)=228
Percent survival
Months

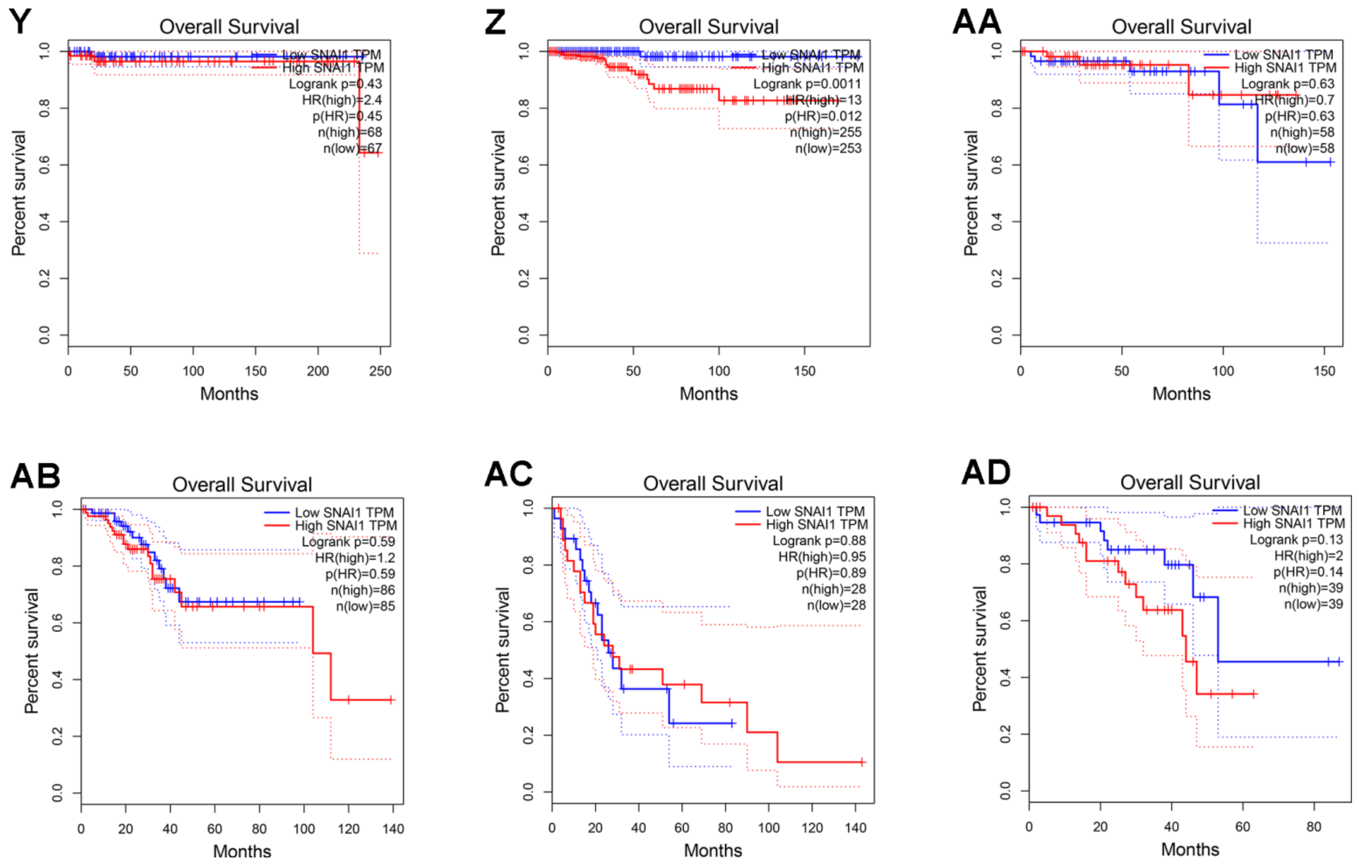

Supplementary Figure 4. Kaplan-Meier survival curves comparing the high and low expression of SNAI1 in different types of human cancers in TCGA dataset.

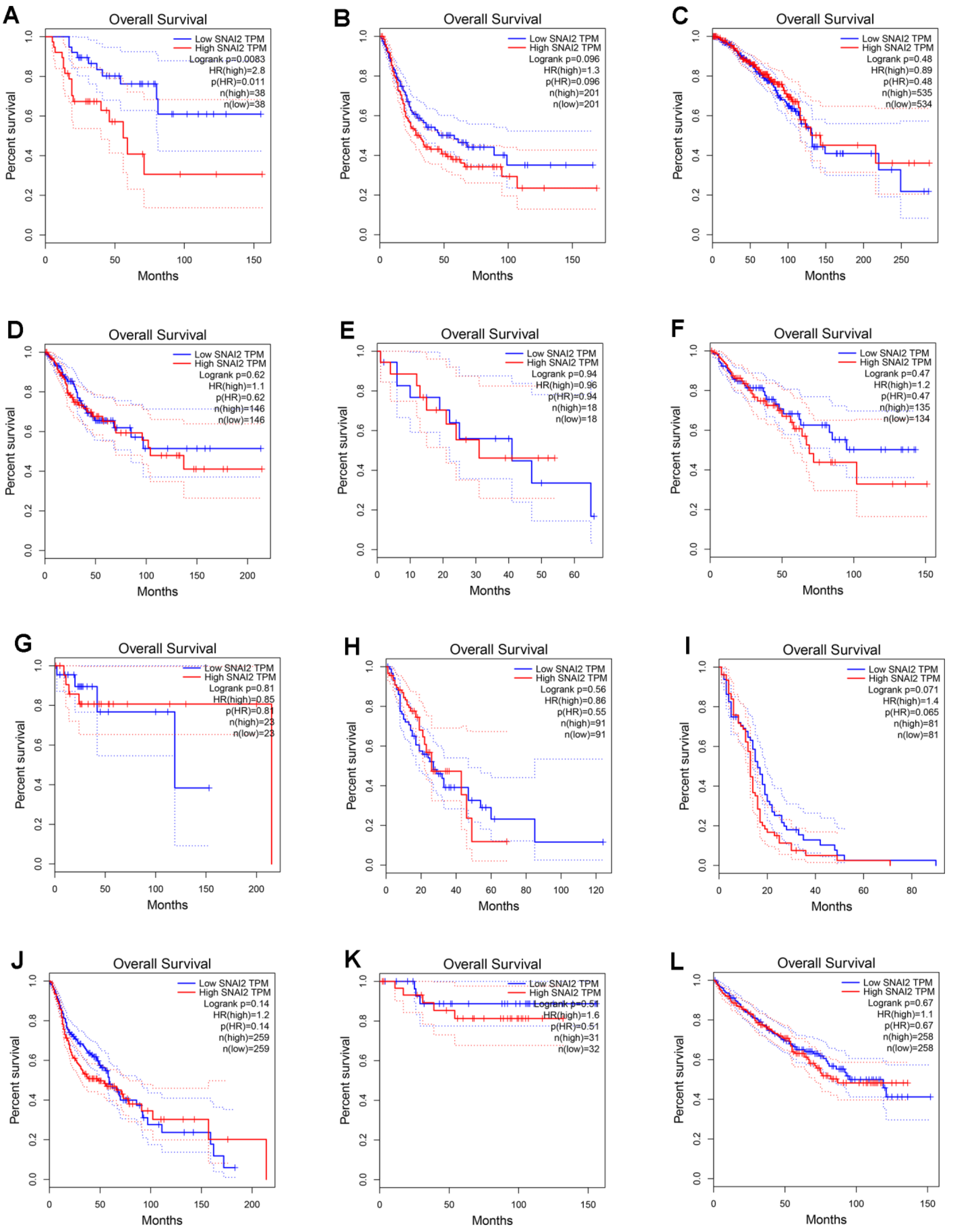
A
Overall Survival
Low SNAI2 TPM
High SNAI2 TPM
Logrank p=0.0083
HR(high)=2.8
p(HR)=0.011
n(high)=38
n(low)=38
Percent survival
Months
B
Overall Survival
Low SNAI2 TPM
High SNAI2 TPM
Logrank p=0.096
HR(high)=1.3
p(HR)=0.096
n(high)=201
n(low)=201
Percent survival
Months
C
Overall Survival
Low SNAI2 TPM
High SNAI2 TPM
Logrank p=0.48
HR(high)=0.89
p(HR)=0.48
n(high)=535
n(low)=534
Percent survival
Months
D
Overall Survival
Low SNAI2 TPM
High SNAI2 TPM
Logrank p=0.62
HR(high)=1.1
p(HR)=0.62
n(high)=146
n(low)=146
Percent survival
Months
E
Overall Survival
Low SNAI2 TPM
High SNAI2 TPM
Logrank p=0.94
HR(high)=0.96
p(HR)=0.94
n(high)=18
n(low)=18
Percent survival
Months
F
Overall Survival
Low SNAI2 TPM
High SNAI2 TPM
Logrank p=0.47
HR(high)=1.2
p(HR)=0.47
n(high)=135
n(low)=134
Percent survival
Months
G
Overall Survival
Low SNAI2 TPM
High SNAI2 TPM
Logrank p=0.81
HR(high)=0.85
p(HR)=0.81
n(high)=23
n(low)=23
Percent survival
Months
H
Overall Survival
Low SNAI2 TPM
High SNAI2 TPM
Logrank p=0.56
HR(high)=0.86
p(HR)=0.55
n(high)=91
n(low)=91
Percent survival
Months
I
Overall Survival
Low SNAI2 TPM
High SNAI2 TPM
Logrank p=0.071
HR(high)=1.4
p(HR)=0.065
n(high)=81
n(low)=81
Percent survival
Months
J
Overall Survival
Low SNAI2 TPM
High SNAI2 TPM
Logrank p=0.14
HR(high)=1.2
p(HR)=0.14
n(high)=259
n(low)=259
Percent survival
Months
K
Overall Survival
Low SNAI2 TPM
High SNAI2 TPM
HR(high)=1.6
p(HR)=0.51
n(high)=31
n(low)=32
Percent survival
Months
L
Overall Survival
Low SNAI2 TPM
High SNAI2 TPM
Logrank p=0.67
HR(high)=1.1
p(HR)=0.67
n(high)=258
n(low)=258
Percent survival
Months

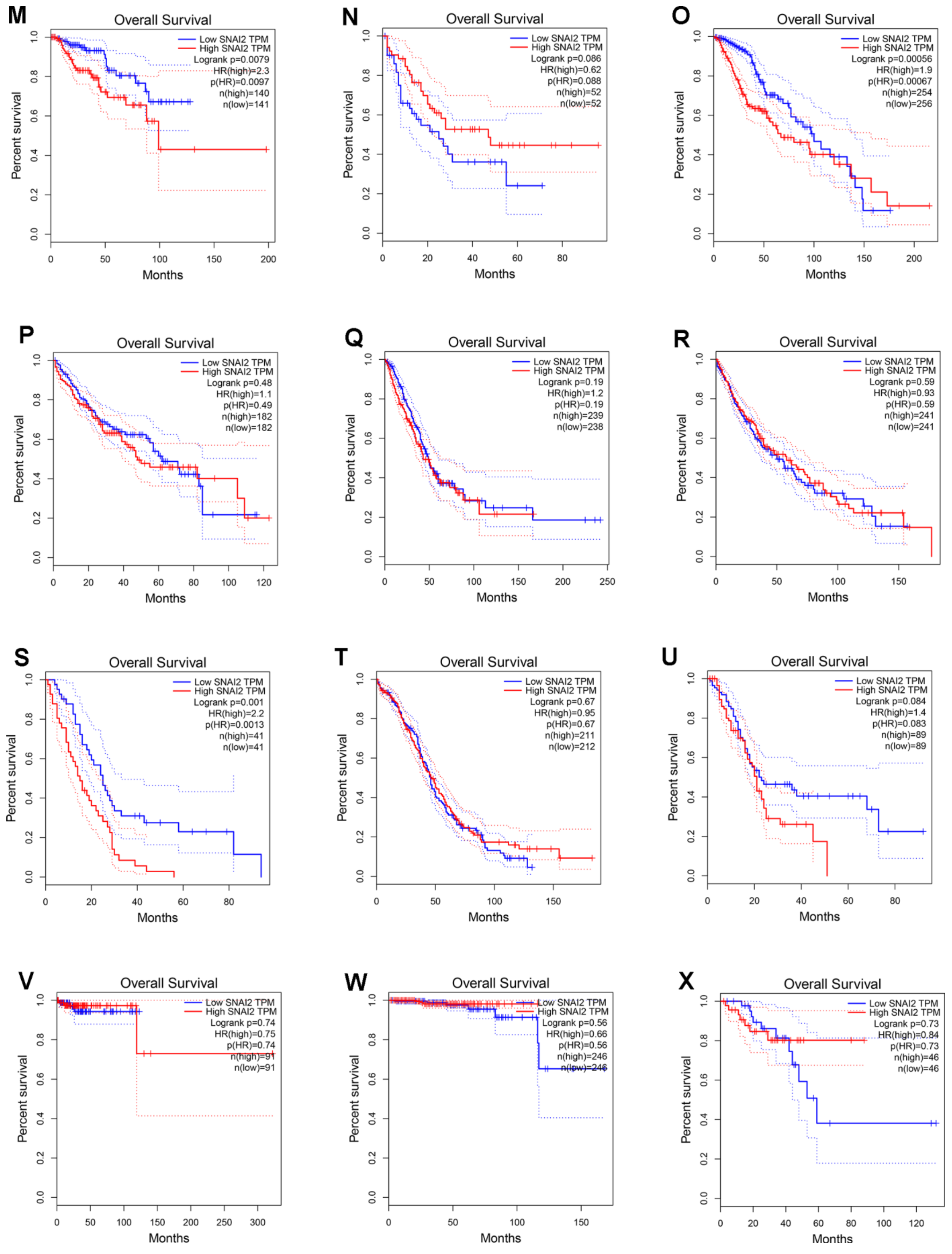

M
Overall Survival
Low SNAI2 TPM
High SNAI2 TPM
Logrank p=0.0079
HR(high)=2.3
p(HR)=0.0097
n(high)=140
n(low)=141
Percent survival
Months
N
Overall Survival
Low SNAI2 TPM
High SNAI2 TPM
Logrank p=0.086
HR(high)=0.62
p(HR)=0.088
n(high)=52
n(low)=52
Percent survival
Months
O
Overall Survival
Low SNAI2 TPM
High SNAI2 TPM
Logrank p=0.00056
HR(high)=1.9
p(HR)=0.00067
n(high)=254
n(low)=256
Percent survival
Months
P
Overall Survival
Low SNAI2 TPM
High SNAI2 TPM
Logrank p=0.48
HR(high)=1.1
p(HR)=0.49
n(high)=182
n(low)=182
Percent survival
Months
Q
Overall Survival
Low SNAI2 TPM
High SNAI2 TPM
Logrank p=0.19
HR(high)=1.2
p(HR)=0.19
n(high)=239
n(low)=238
Percent survival
Months
R
Overall Survival
Low SNAI2 TPM
High SNAI2 TPM
Logrank p=0.59
HR(high)=0.93
p(HR)=0.59
n(high)=241
n(low)=241
Percent survival
Months
S
Overall Survival
Low SNAI2 TPM
High SNAI2 TPM
Logrank p=0.001
HR(high)=2.2
p(HR)=0.0013
n(high)=41
n(low)=41
Percent survival
Months
T
Overall Survival
Low SNAI2 TPM
High SNAI2 TPM
Logrank p=0.67
HR(high)=0.95
p(HR)=0.67
n(high)=211
n(low)=212
Percent survival
Months
U
Overall Survival
Low SNAI2 TPM
High SNAI2 TPM
Logrank p=0.084
HR(high)=1.4
p(HR)=0.083
n(high)=89
n(low)=89
Percent survival
Months
V
Overall Survival
Low SNAI2 TPM
High SNAI2 TPM
Logrank p=0.74
HR(high)=0.75
p(HR)=0.74
n(high)=91
n(low)=91
Percent survival
Months
W
Overall Survival
Low SNAI2 TPM
High SNAI2 TPM
Logrank p=0.56
HR(high)=0.66
p(HR)=0.56
n(high)=246
n(low)=246
Percent survival
Months
X
Overall Survival
Low SNAI2 TPM
High SNAI2 TPM
Logrank p=0.73
HR(high)=0.84
p(HR)=0.73
n(high)=46
n(low)=46
Percent survival
Months

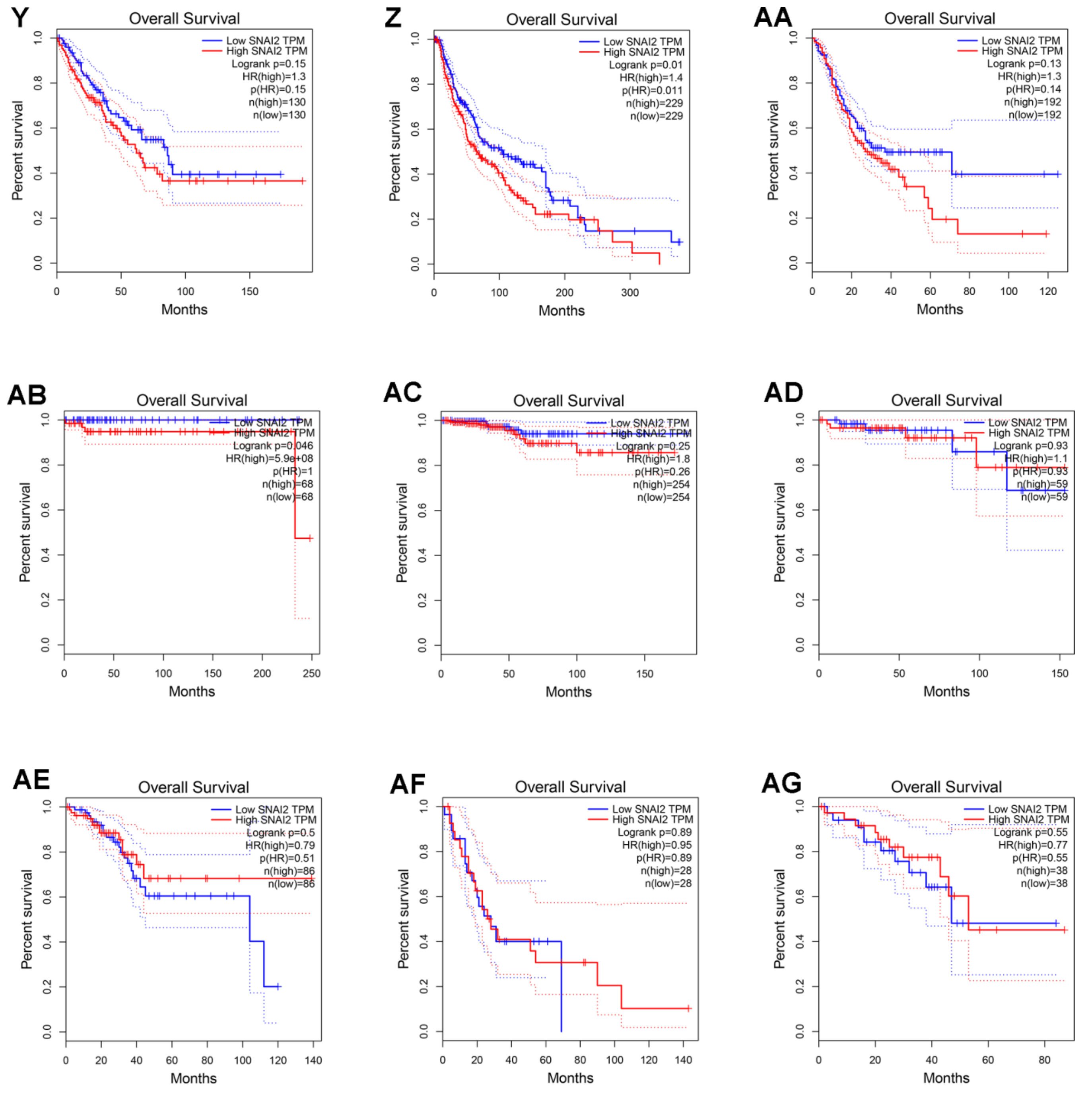

Supplementary Figure 5. Kaplan-Meier survival curves comparing the high and low expression of SNAI2 in different types of human cancers in TCGA dataset.

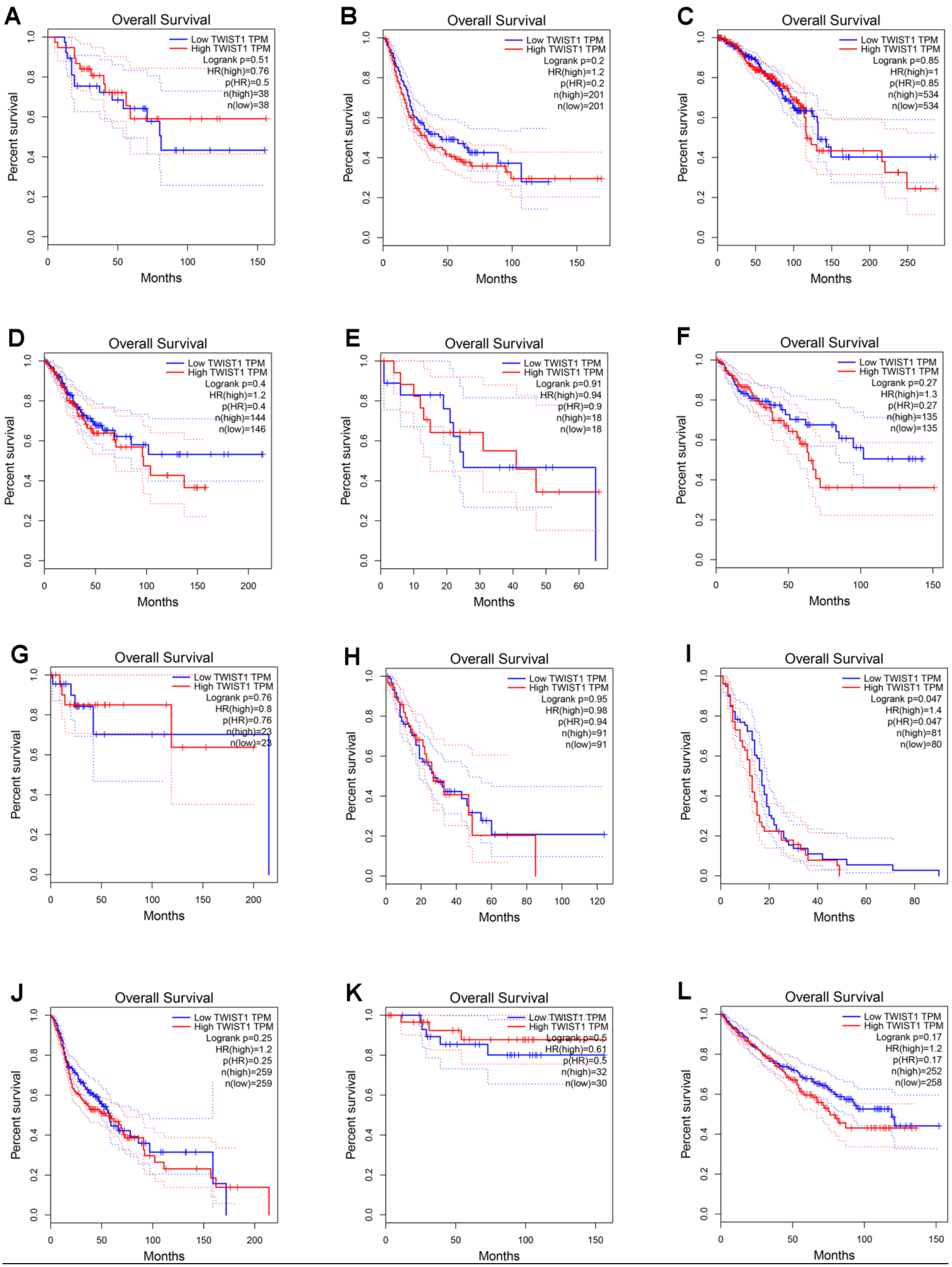
A
Overall Survival
Low TWIST1 TPM
High TWIST1 TPM
Logrank p=0.51
HR(high)=0.76
p(HR)=0.5
n(high)=38
n(low)=38
B
Overall Survival
Low TWIST1 TPM
High TWIST1 TPM
Logrank p=0.2
HR(high)=1.2
p(HR)=0.2
n(high)=201
n(low)=201
C
Overall Survival
Low TWIST1 TPM
High TWIST1 TPM
Logrank p=0.85
HR(high)=1
p(HR)=0.85
n(high)=534
n(low)=534
D
Overall Survival
Low TWIST1 TPM
High TWIST1 TPM
Logrank p=0.4
HR(high)=1.2
p(HR)=0.4
n(high)=144
n(low)=146
E
Overall Survival
Low TWIST1 TPM
High TWIST1 TPM
Logrank p=0.91
HR(high)=0.94
p(HR)=0.9
n(high)=18
n(low)=18
F
Overall Survival
Low TWIST1 TPM
High TWIST1 TPM
Logrank p=0.27
HR(high)=1.3
p(HR)=0.27
n(high)=135
n(low)=135
G
Overall Survival
Low TWIST1 TPM
High TWIST1 TPM
Logrank p=0.76
HR(high)=0.8
p(HR)=0.76
n(high)=23
n(low)=23
H
Overall Survival
Low TWIST1 TPM
High TWIST1 TPM
Logrank p=0.95
HR(high)=0.98
p(HR)=0.94
n(high)=91
n(low)=91
I
Overall Survival
Low TWIST1 TPM
High TWIST1 TPM
Logrank p=0.047
HR(high)=1.4
p(HR)=0.047
n(high)=81
n(low)=80
J
Overall Survival
Low TWIST1 TPM
High TWIST1 TPM
Logrank p=0.25
HR(high)=1.2
p(HR)=0.25
n(high)=259
n(low)=259
K
Overall Survival
Low TWIST1 TPM
High TWIST1 TPM
Logrank p=0.5
HR(high)=0.61
p(HR)=0.5
n(high)=32
n(low)=30
L
Overall Survival
Low TWIST1 TPM
High TWIST1 TPM
Logrank p=0.17
HR(high)=1.2
p(HR)=0.17
n(high)=252
n(low)=258
Percent survival
Months

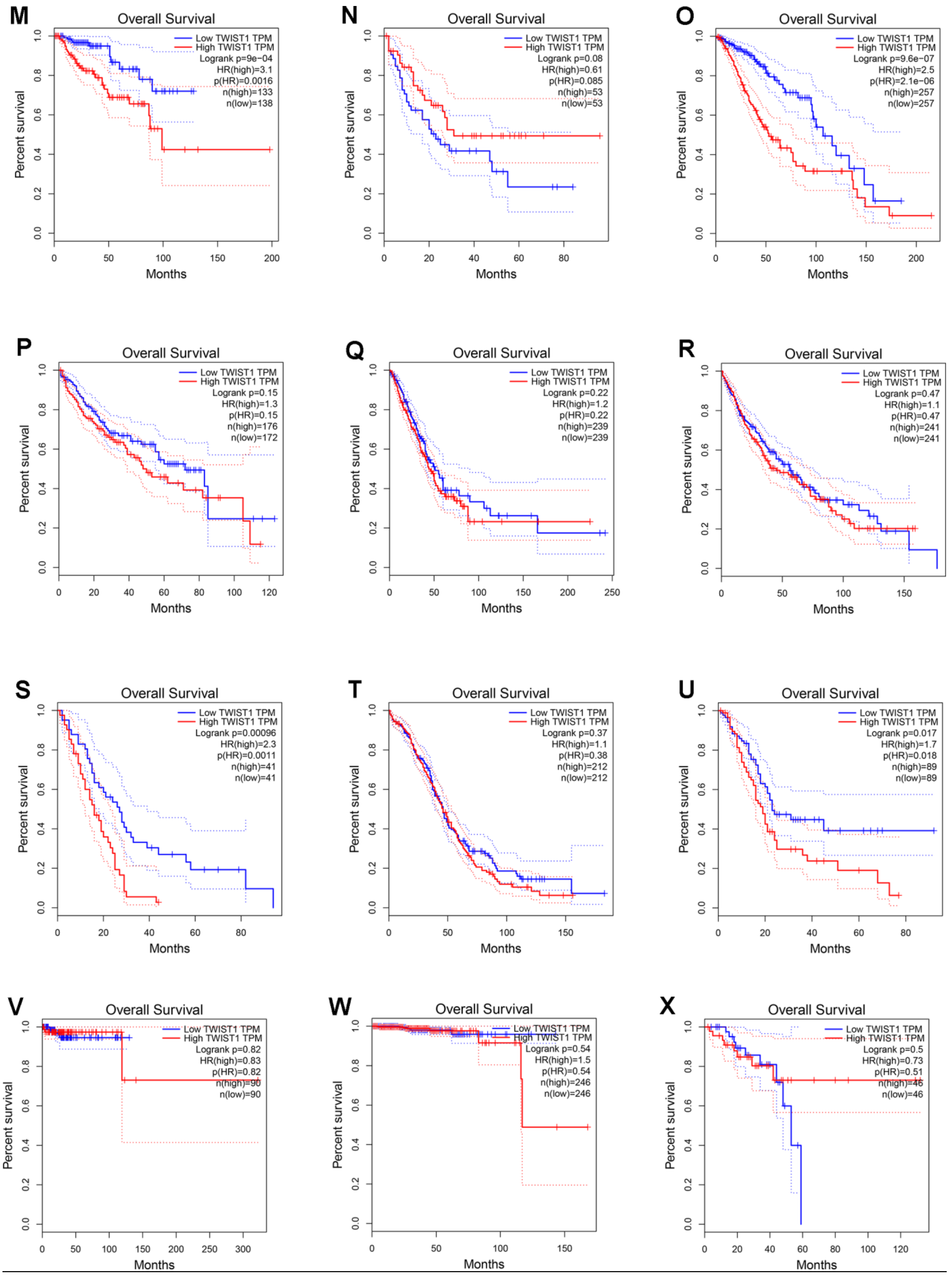
M
Overall Survival
Low TWIST1 TPM
High TWIST1 TPM
Logrank p=9e−04
HR(high)=3.1
p(HR)=0.0016
n(high)=133
n(low)=138
N
Overall Survival
Low TWIST1 TPM
High TWIST1 TPM
Logrank p=0.08
HR(high)=0.61
p(HR)=0.085
n(high)=53
n(low)=53
O
Overall Survival
Low TWIST1 TPM
High TWIST1 TPM
Logrank p=9.6e−07
HR(high)=2.5
p(HR)=2.1e−06
n(high)=257
n(low)=257
P
Overall Survival
Low TWIST1 TPM
High TWIST1 TPM
Logrank p=0.15
HR(high)=1.3
p(HR)=0.15
n(high)=176
n(low)=172
Q
Overall Survival
Low TWIST1 TPM
High TWIST1 TPM
Logrank p=0.22
HR(high)=1.2
p(HR)=0.22
n(high)=239
n(low)=239
R
Overall Survival
Low TWIST1 TPM
High TWIST1 TPM
Logrank p=0.47
HR(high)=1.1
p(HR)=0.47
n(high)=241
n(low)=241
S
Overall Survival
Low TWIST1 TPM
High TWIST1 TPM
Logrank p=0.00096
HR(high)=2.3
p(HR)=0.0011
n(high)=41
n(low)=41
T
Overall Survival
Low TWIST1 TPM
High TWIST1 TPM
Logrank p=0.37
HR(high)=1.1
p(HR)=0.38
n(high)=212
n(low)=212
U
Overall Survival
Low TWIST1 TPM
High TWIST1 TPM
Logrank p=0.017
HR(high)=1.7
p(HR)=0.018
n(high)=89
n(low)=89
V
Overall Survival
Low TWIST1 TPM
High TWIST1 TPM
Logrank p=0.82
HR(high)=0.83
p(HR)=0.82
n(high)=90
n(low)=90
W
Overall Survival
Low TWIST1 TPM
High TWIST1 TPM
Logrank p=0.54
HR(high)=1.5
p(HR)=0.54
n(high)=246
n(low)=246
X
Overall Survival
Low TWIST1 TPM
High TWIST1 TPM
Logrank p=0.5
HR(high)=0.73
p(HR)=0.51
n(high)=46
n(low)=46
Percent survival
Months

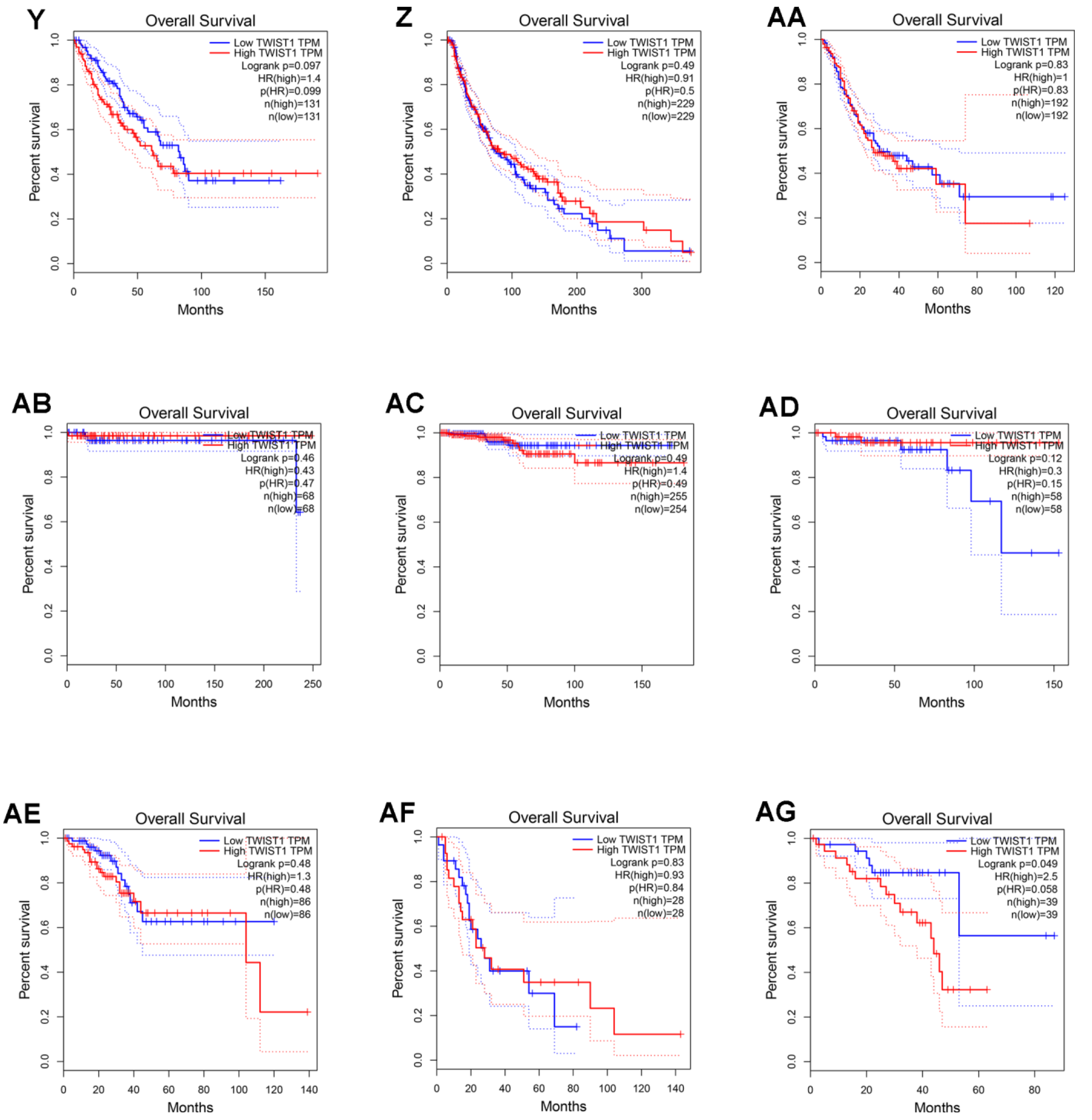

Supplementary Figure 6. Kaplan-Meier survival curves comparing the high and low expression of TWIST1 in different types of human cancers in TCGA dataset.

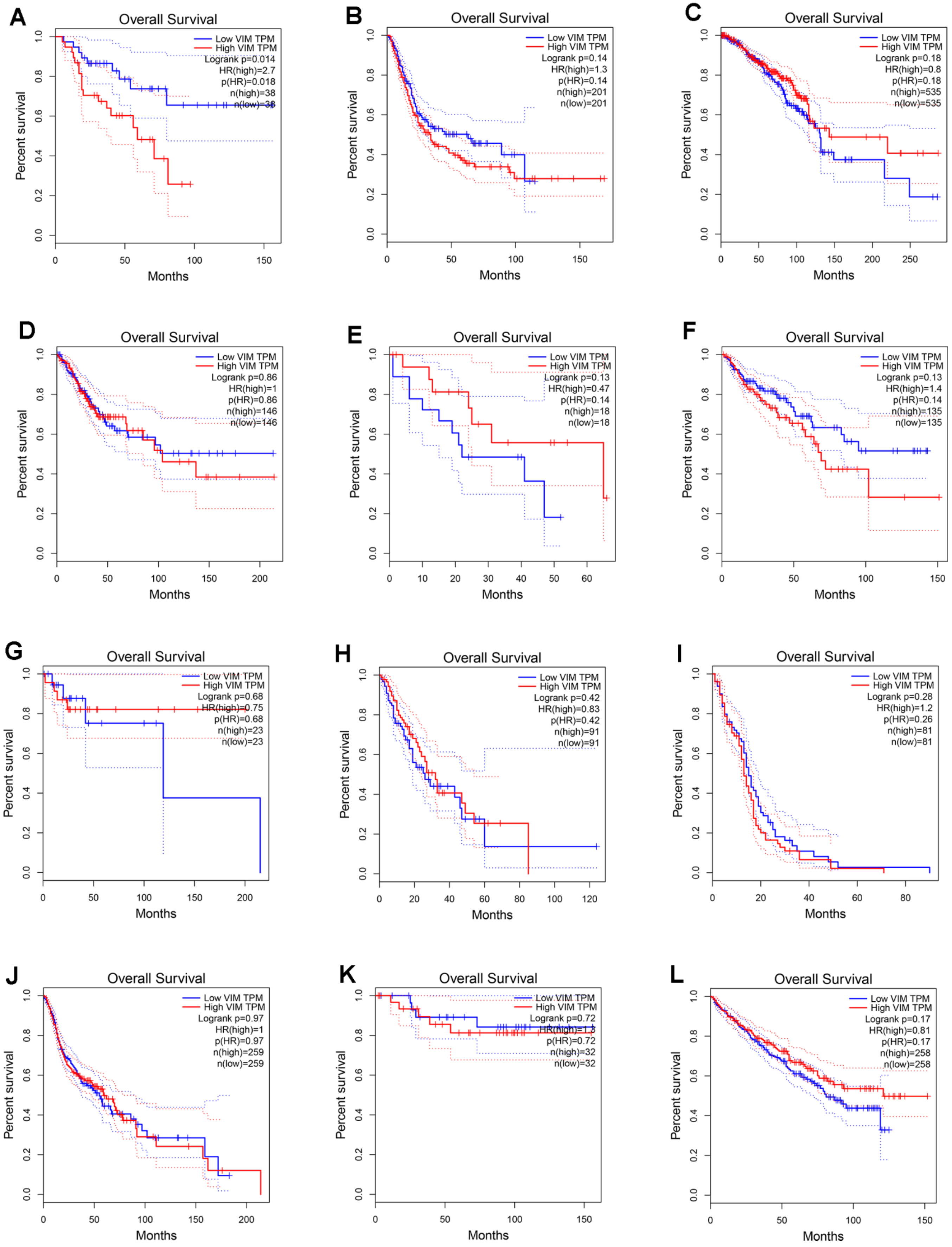
A
Overall Survival
Low VIM TPM
High VIM TPM
Logrank p=0.014
HR(high)=2.7
p(HR)=0.018
n(high)=38
n(low)=38
B
Overall Survival
Low VIM TPM
High VIM TPM
Logrank p=0.14
HR(high)=1.3
p(HR)=0.14
n(high)=201
n(low)=201
C
Overall Survival
Low VIM TPM
High VIM TPM
Logrank p=0.18
HR(high)=0.8
p(HR)=0.18
n(high)=535
n(low)=535
D
Overall Survival
Low VIM TPM
High VIM TPM
Logrank p=0.86
HR(high)=1
p(HR)=0.86
n(high)=146
n(low)=146
E
Overall Survival
Low VIM TPM
High VIM TPM
Logrank p=0.13
HR(high)=0.47
p(HR)=0.14
n(high)=18
n(low)=18
F
Overall Survival
Low VIM TPM
High VIM TPM
Logrank p=0.13
HR(high)=1.4
p(HR)=0.14
n(high)=135
n(low)=135
G
Overall Survival
Low VIM TPM
High VIM TPM
Logrank p=0.68
HR(high)=0.75
p(HR)=0.68
n(high)=23
n(low)=23
H
Overall Survival
Low VIM TPM
High VIM TPM
Logrank p=0.42
HR(high)=0.83
p(HR)=0.42
n(high)=91
n(low)=91
I
Overall Survival
Low VIM TPM
High VIM TPM
Logrank p=0.28
HR(high)=1.2
p(HR)=0.26
n(high)=81
n(low)=81
J
Overall Survival
Low VIM TPM
High VIM TPM
Logrank p=0.97
HR(high)=1
p(HR)=0.97
n(high)=259
n(low)=259
K
Overall Survival
Low VIM TPM
High VIM TPM
Logrank p=0.72
HR(high)=1.3
p(HR)=0.72
n(high)=32
n(low)=32
L
Overall Survival
Low VIM TPM
High VIM TPM
Logrank p=0.17
HR(high)=0.81
p(HR)=0.17
n(high)=258
n(low)=258
Percent survival
Months

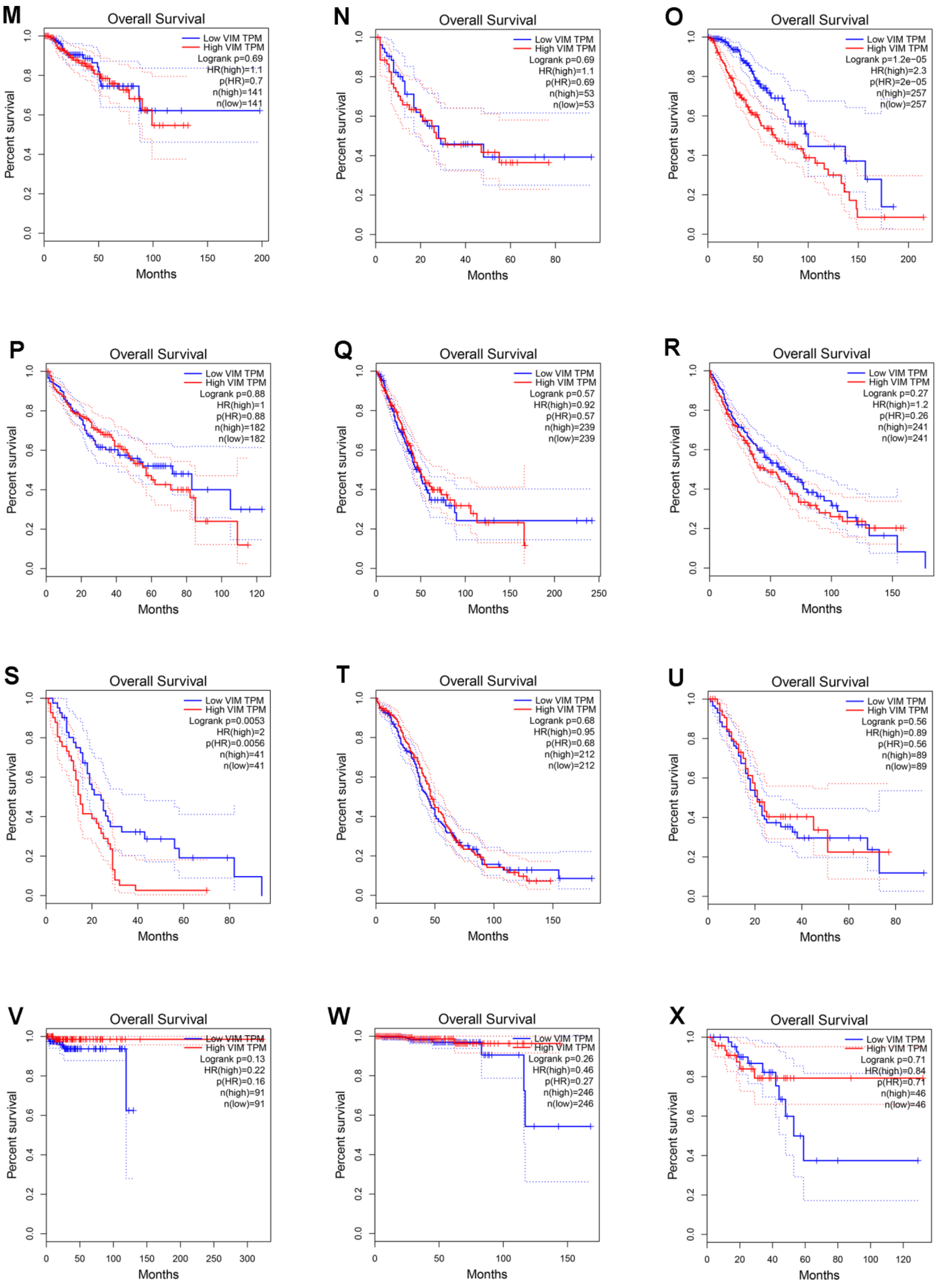
M
Overall Survival
Low VIM TPM
High VIM TPM
Logrank p=0.69
HR(high)=1.1
p(HR)=0.7
n(high)=141
n(low)=141
Percent survival
Months
N
Overall Survival
Low VIM TPM
High VIM TPM
Logrank p=0.69
HR(high)=1.1
p(HR)=0.69
n(high)=53
n(low)=53
Percent survival
Months
O
Overall Survival
Low VIM TPM
High VIM TPM
Logrank p=1.2e−05
HR(high)=2.3
p(HR)=2e−05
n(high)=257
n(low)=257
Percent survival
Months
P
Overall Survival
Low VIM TPM
High VIM TPM
Logrank p=0.88
HR(high)=1
p(HR)=0.88
n(high)=182
n(low)=182
Percent survival
Months
Q
Overall Survival
Low VIM TPM
High VIM TPM
Logrank p=0.57
HR(high)=0.92
p(HR)=0.57
n(high)=239
n(low)=239
Percent survival
Months
R
Overall Survival
Low VIM TPM
High VIM TPM
Logrank p=0.27
HR(high)=1.2
p(HR)=0.26
n(high)=241
n(low)=241
Percent survival
Months
S
Overall Survival
Low VIM TPM
High VIM TPM
Logrank p=0.0053
HR(high)=2
p(HR)=0.0056
n(high)=41
n(low)=41
Percent survival
Months
T
Overall Survival
Low VIM TPM
High VIM TPM
Logrank p=0.68
HR(high)=0.95
p(HR)=0.68
n(high)=212
n(low)=212
Percent survival
Months
U
Overall Survival
Low VIM TPM
High VIM TPM
Logrank p=0.56
HR(high)=0.89
p(HR)=0.56
n(high)=89
n(low)=89
Percent survival
Months
V
Overall Survival
Low VIM TPM
High VIM TPM
Logrank p=0.13
HR(high)=0.22
p(HR)=0.16
n(high)=91
n(low)=91
Percent survival
Months
W
Overall Survival
Low VIM TPM
High VIM TPM
Logrank p=0.26
HR(high)=0.46
p(HR)=0.27
n(high)=246
n(low)=246
Percent survival
Months
X
Overall Survival
Low VIM TPM
High VIM TPM
Logrank p=0.71
HR(high)=0.84
p(HR)=0.71
n(high)=46
n(low)=46
Percent survival
Months

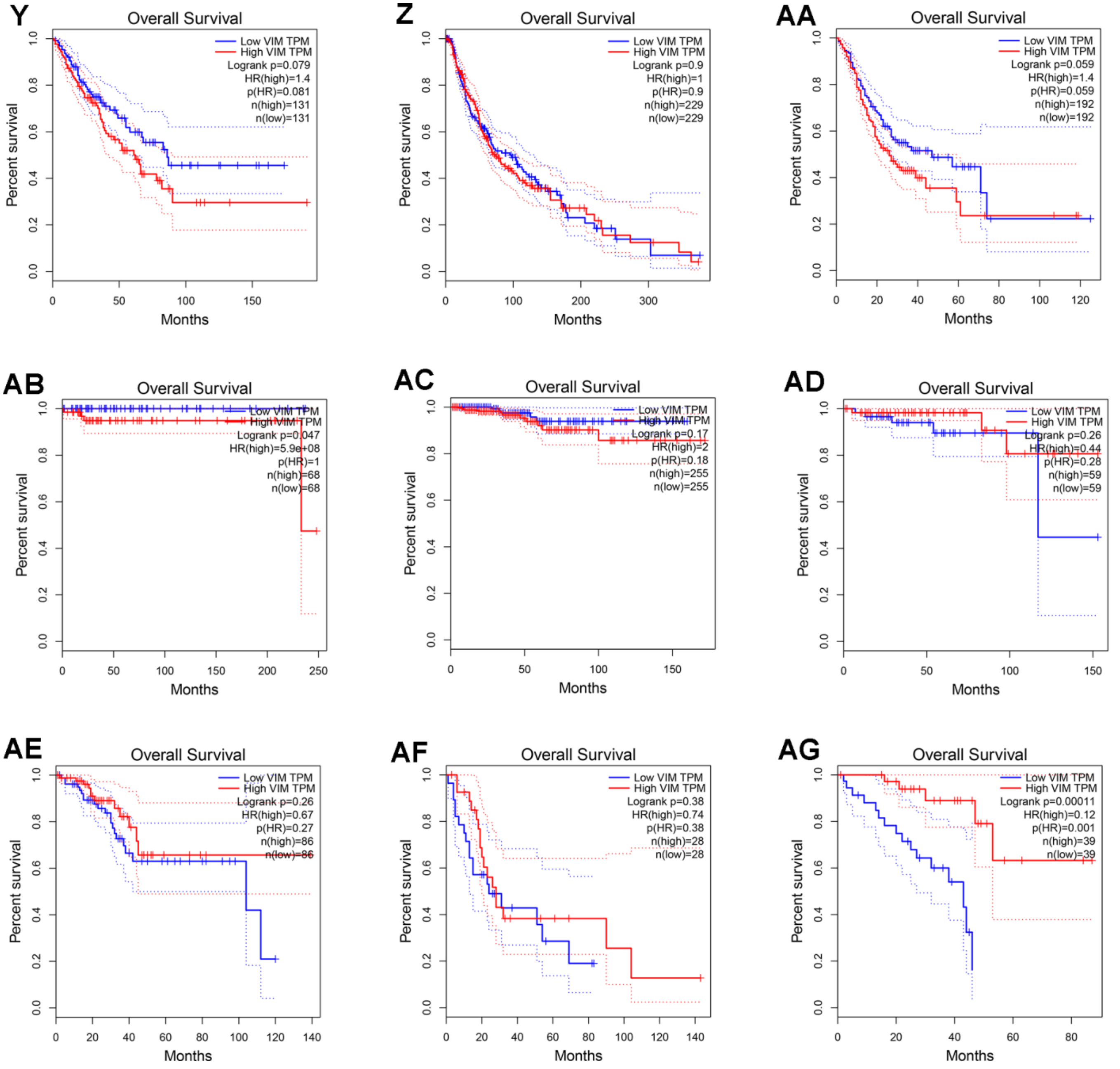

Supplementary Figure 7. Kaplan-Meier survival curves comparing the high and low expression of VIM in different types of human cancers in TCGA dataset.

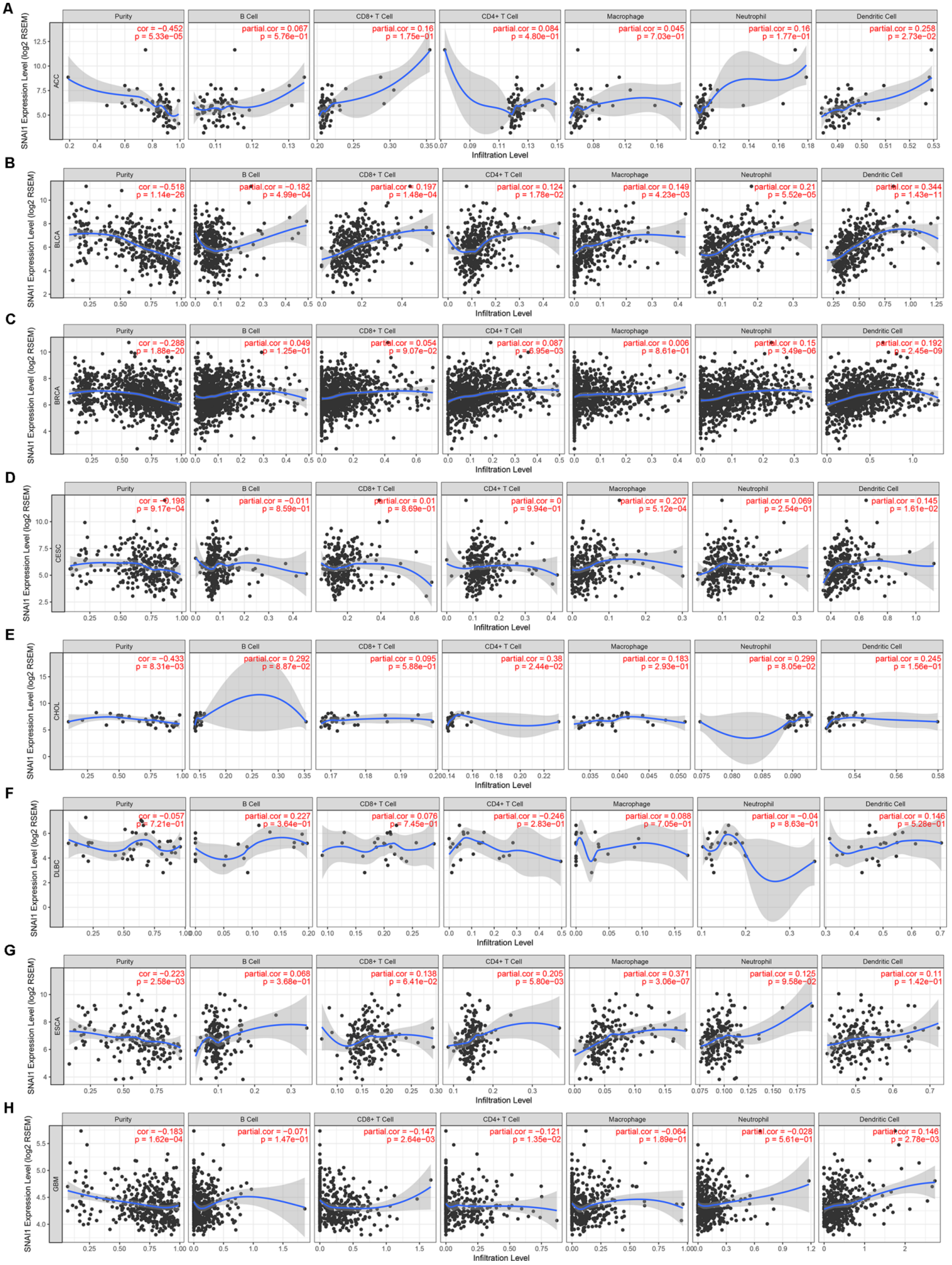
SNAI1 Expression Level (log2 RSEM)
Infiltration Level
Purity
B Cell
CD8+ T Cell
CD4+ T Cell
Macrophage
Neutrophil
Dendritic Cell
A
ACC
cor = −0.452 p = 5.33e−05
partial.cor = 0.067 p = 5.76e−01
partial.cor = 0.16 p = 1.75e−01
partial.cor = 0.084 p = 4.80e−01
partial.cor = 0.045 p = 7.03e−01
partial.cor = 0.16 p = 1.77e−01
partial.cor = 0.258 p = 2.73e−02
B
BLCA
cor = −0.518 p = 1.14e−26
partial.cor = −0.182 p = 4.99e−04
partial.cor = 0.197 p = 1.48e−04
partial.cor = 0.124 p = 1.78e−02
partial.cor = 0.149 p = 4.23e−03
partial.cor = 0.21 p = 5.52e−05
partial.cor = 0.344 p = 1.43e−11
C
BRCA
cor = −0.288 p = 1.88e−20
partial.cor = 0.049 p = 1.25e−01
partial.cor = 0.054 p = 9.07e−02
partial.cor = 0.087 p = 6.95e−03
partial.cor = 0.006 p = 8.61e−01
partial.cor = 0.15 p = 3.49e−06
partial.cor = 0.192 p = 2.45e−09
D
CESC
cor = −0.198 p = 9.17e−04
partial.cor = −0.011 p = 8.59e−01
partial.cor = 0.01 p = 8.69e−01
partial.cor = 0 p = 9.94e−01
partial.cor = 0.207 p = 5.12e−04
partial.cor = 0.069 p = 2.54e−01
partial.cor = 0.145 p = 1.61e−02
E
CHOL
cor = −0.433 p = 8.31e−03
partial.cor = 0.292 p = 8.87e−02
partial.cor = 0.095 p = 5.88e−01
partial.cor = 0.38 p = 2.44e−02
partial.cor = 0.183 p = 2.93e−01
partial.cor = 0.299 p = 8.05e−02
partial.cor = 0.245 p = 1.56e−01
F
DLBC
cor = −0.057 p = 7.21e−01
partial.cor = 0.227 p = 3.64e−01
partial.cor = 0.076 p = 7.45e−01
partial.cor = −0.246 p = 2.83e−01
partial.cor = 0.088 p = 7.05e−01
partial.cor = −0.04 p = 8.63e−01
partial.cor = 0.146 p = 5.28e−01
G
ESCA
cor = −0.223 p = 2.58e−03
partial.cor = 0.068 p = 3.68e−01
partial.cor = 0.138 p = 6.41e−02
partial.cor = 0.205 p = 5.80e−03
partial.cor = 0.371 p = 3.06e−07
partial.cor = 0.125 p = 9.58e−02
partial.cor = 0.11 p = 1.42e−01
H
GBM
cor = −0.183 p = 1.62e−04
partial.cor = −0.071 p = 1.47e−01
partial.cor = −0.147 p = 2.64e−03
partial.cor = −0.121 p = 1.35e−02
partial.cor = −0.064 p = 1.89e−01
partial.cor = −0.028 p = 5.61e−01
partial.cor = 0.146 p = 2.78e−03

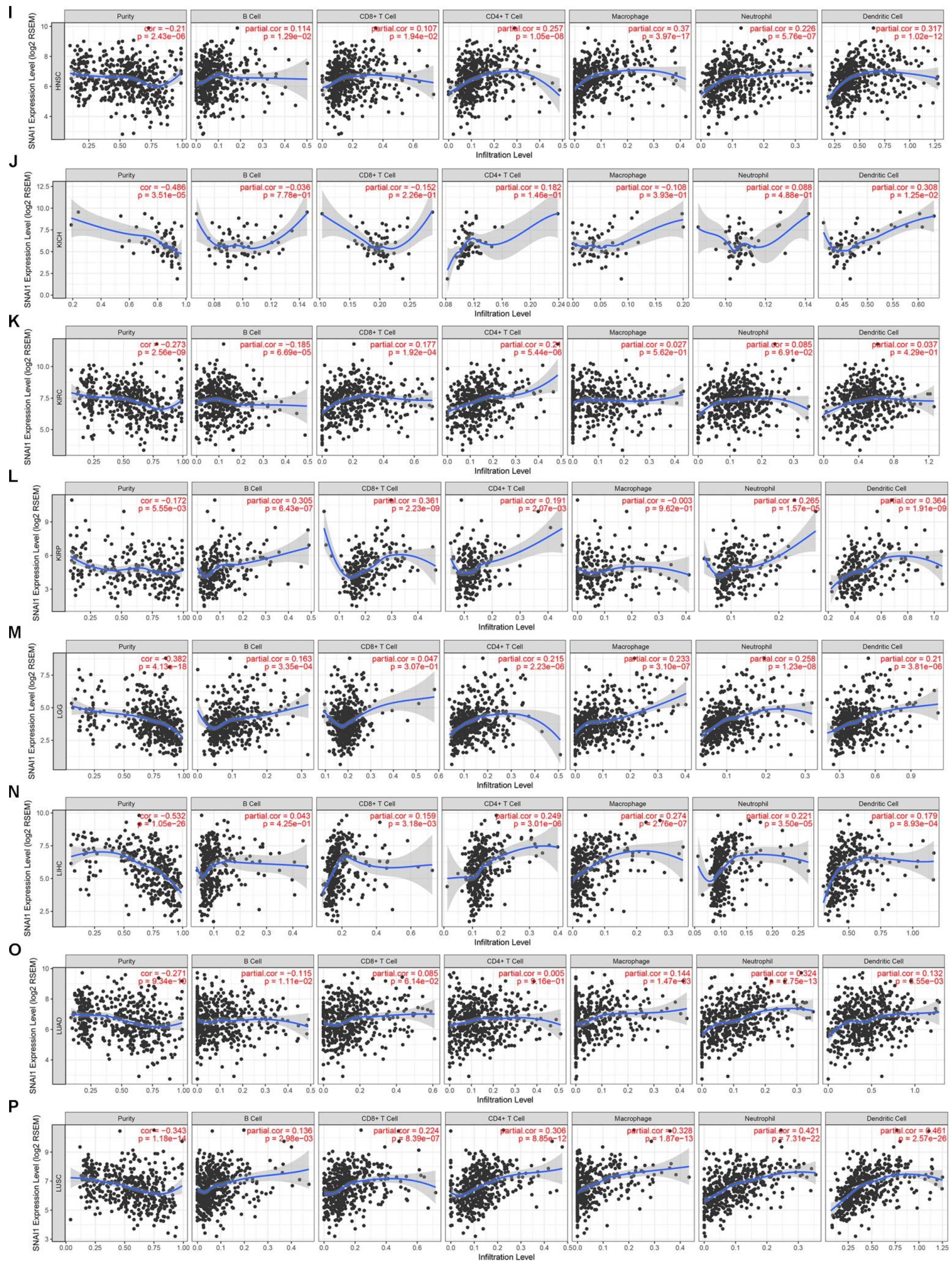

I
J
K
L
M
N
O
P
SNAI1 Expression Level (log2 RSEM)
Infiltration Level
Purity
B Cell
CD8+ T Cell
CD4+ T Cell
Macrophage
Neutrophil
Dendritic Cell
HNSC
KICH
KIRC
KIRP
LGG
LIHC
LUAD
LUSC

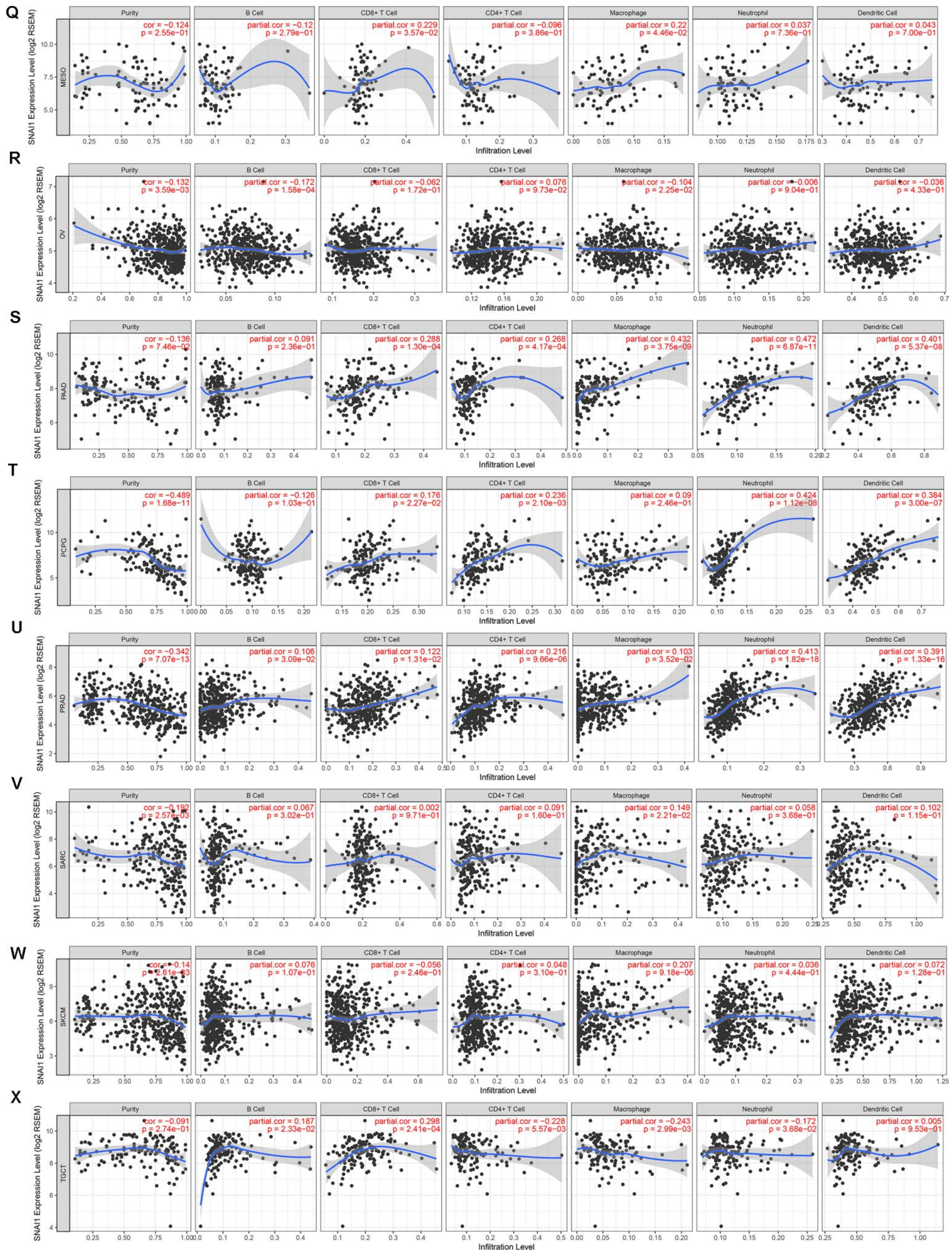
Q
R
S
T
U
V
W
X
Purity
B Cell
CD8+ T Cell
CD4+ T Cell
Macrophage
Neutrophil
Dendritic Cell
SNAI1 Expression Level (log2 RSEM)
Infiltration Level
MESO
cor = −0.124 p = 2.55e−01
partial.cor = −0.12 p = 2.79e−01
partial.cor = 0.229 p = 3.57e−02
partial.cor = −0.096 p = 3.86e−01
partial.cor = 0.22 p = 4.46e−02
partial.cor = 0.037 p = 7.36e−01
partial.cor = 0.043 p = 7.00e−01
OV
cor = −0.132 p = 3.59e−03
partial.cor = −0.172 p = 1.58e−04
partial.cor = −0.062 p = 1.72e−01
partial.cor = 0.076 p = 9.73e−02
partial.cor = −0.104 p = 2.25e−02
partial.cor = −0.006 p = 9.04e−01
partial.cor = −0.036 p = 4.33e−01
PAAD
cor = −0.136 p = 7.46e−02
partial.cor = 0.091 p = 2.36e−01
partial.cor = 0.288 p = 1.30e−04
partial.cor = 0.268 p = 4.17e−04
partial.cor = 0.432 p = 3.75e−09
partial.cor = 0.472 p = 6.87e−11
partial.cor = 0.401 p = 5.37e−08
PCPG
cor = −0.489 p = 1.68e−11
partial.cor = −0.126 p = 1.03e−01
partial.cor = 0.176 p = 2.27e−02
partial.cor = 0.236 p = 2.10e−03
partial.cor = 0.09 p = 2.46e−01
partial.cor = 0.424 p = 1.12e−08
partial.cor = 0.384 p = 3.00e−07
PRAD
cor = −0.342 p = 7.07e−13
partial.cor = 0.106 p = 3.09e−02
partial.cor = 0.122 p = 1.31e−02
partial.cor = 0.216 p = 9.66e−06
partial.cor = 0.103 p = 3.52e−02
partial.cor = 0.413 p = 1.82e−18
partial.cor = 0.391 p = 1.33e−16
SARC
cor = −0.192 p = 2.57e−03
partial.cor = 0.067 p = 3.02e−01
partial.cor = 0.002 p = 9.71e−01
partial.cor = 0.091 p = 1.60e−01
partial.cor = 0.149 p = 2.21e−02
partial.cor = 0.058 p = 3.68e−01
partial.cor = 0.102 p = 1.15e−01
SKCM
cor = −0.14 p = 2.61e−03
partial.cor = 0.076 p = 1.07e−01
partial.cor = −0.056 p = 2.46e−01
partial.cor = 0.048 p = 3.10e−01
partial.cor = 0.207 p = 9.18e−06
partial.cor = 0.036 p = 4.44e−01
partial.cor = 0.072 p = 1.28e−01
TGCT
cor = −0.091 p = 2.74e−01
partial.cor = 0.187 p = 2.33e−02
partial.cor = 0.298 p = 2.41e−04
partial.cor = −0.228 p = 5.57e−03
partial.cor = −0.243 p = 2.99e−03
partial.cor = −0.172 p = 3.68e−02
partial.cor = 0.005 p = 9.53e−01

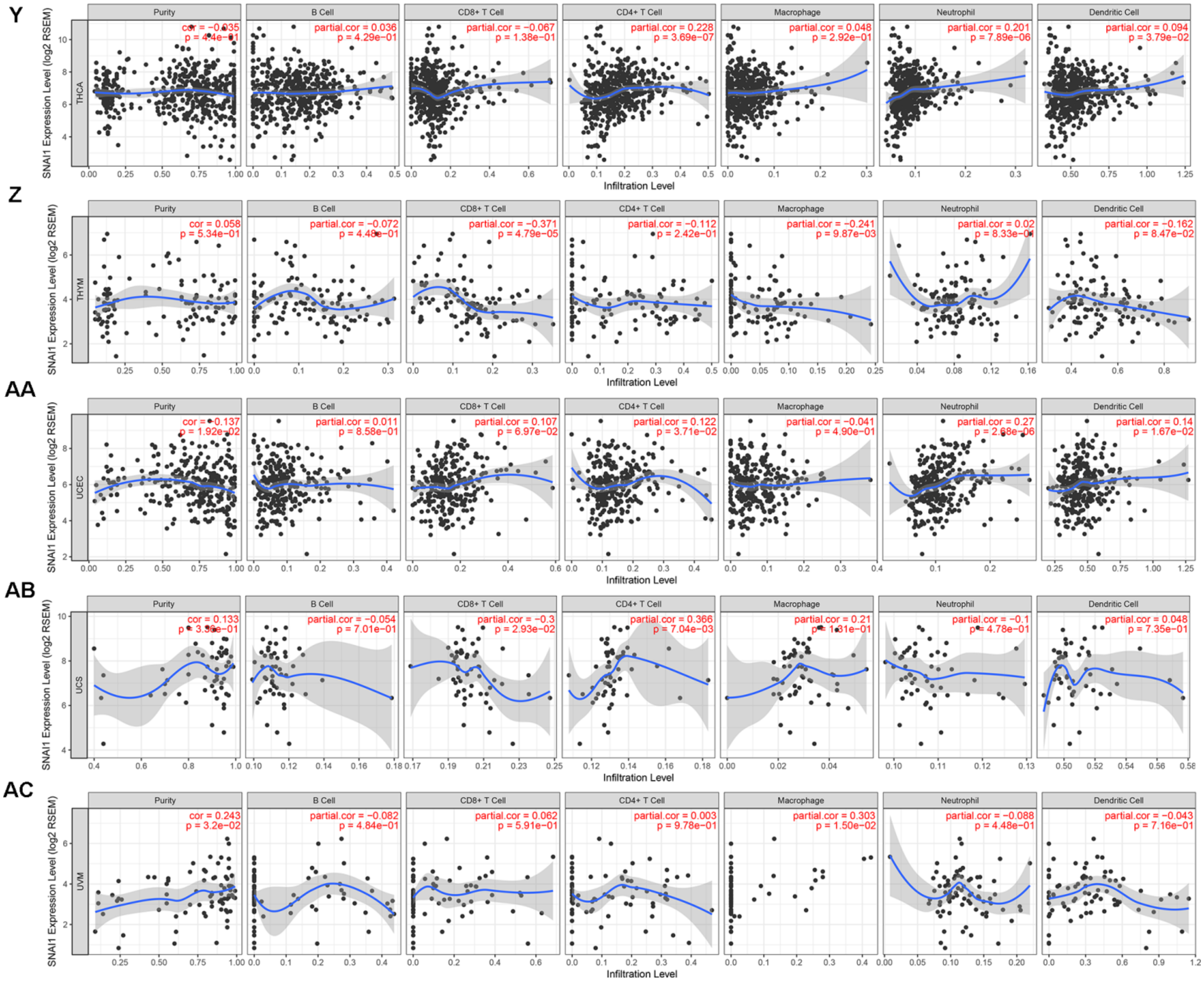

Supplementary Figure 8. Correlation of SNAI1 expression with tumor-infiltrating lymphocytes in different types of human cancers in TCGA dataset.

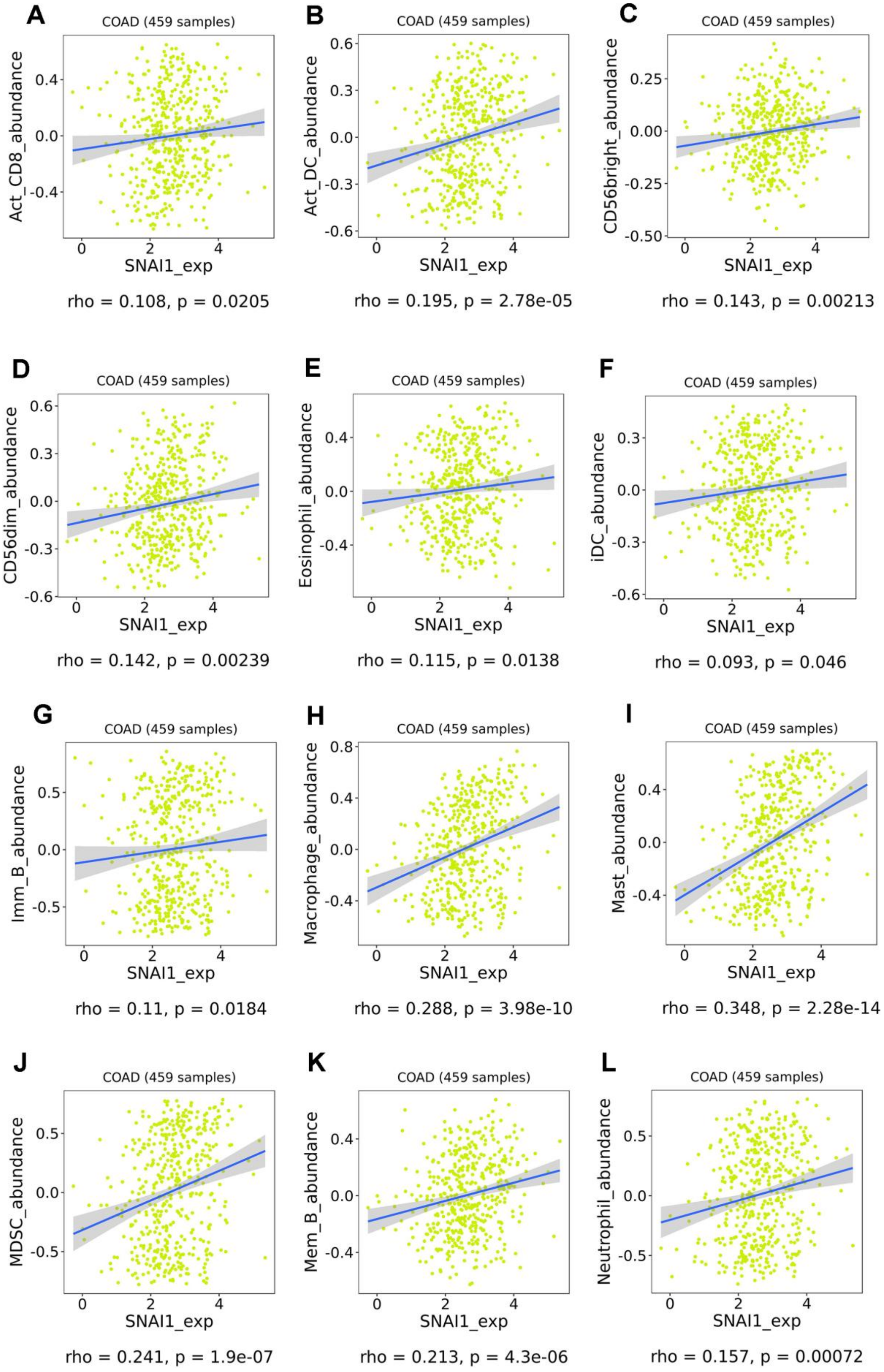
A
COAD (459 samples)
Act_CD8_abundance
SNAI1_exp
rho = 0.108, p = 0.0205
B
COAD (459 samples)
Act_DC_abundance
SNAI1_exp
rho = 0.195, p = 2.78e-05
C
COAD (459 samples)
CD56bright_abundance
SNAI1_exp
rho = 0.143, p = 0.00213
D
COAD (459 samples)
CD56dim_abundance
SNAI1_exp
rho = 0.142, p = 0.00239
E
COAD (459 samples)
Eosinophil_abundance
SNAI1_exp
rho = 0.115, p = 0.0138
F
COAD (459 samples)
iDC_abundance
SNAI1_exp
rho = 0.093, p = 0.046
G
COAD (459 samples)
Imm_B_abundance
SNAI1_exp
rho = 0.11, p = 0.0184
H
COAD (459 samples)
Macrophage_abundance
SNAI1_exp
rho = 0.288, p = 3.98e-10
I
COAD (459 samples)
Mast_abundance
SNAI1_exp
rho = 0.348, p = 2.28e-14
J
COAD (459 samples)
MDSC_abundance
SNAI1_exp
rho = 0.241, p = 1.9e-07
K
COAD (459 samples)
Mem_B_abundance
SNAI1_exp
rho = 0.213, p = 4.3e-06
L
COAD (459 samples)
Neutrophil_abundance
SNAI1_exp
rho = 0.157, p = 0.00072

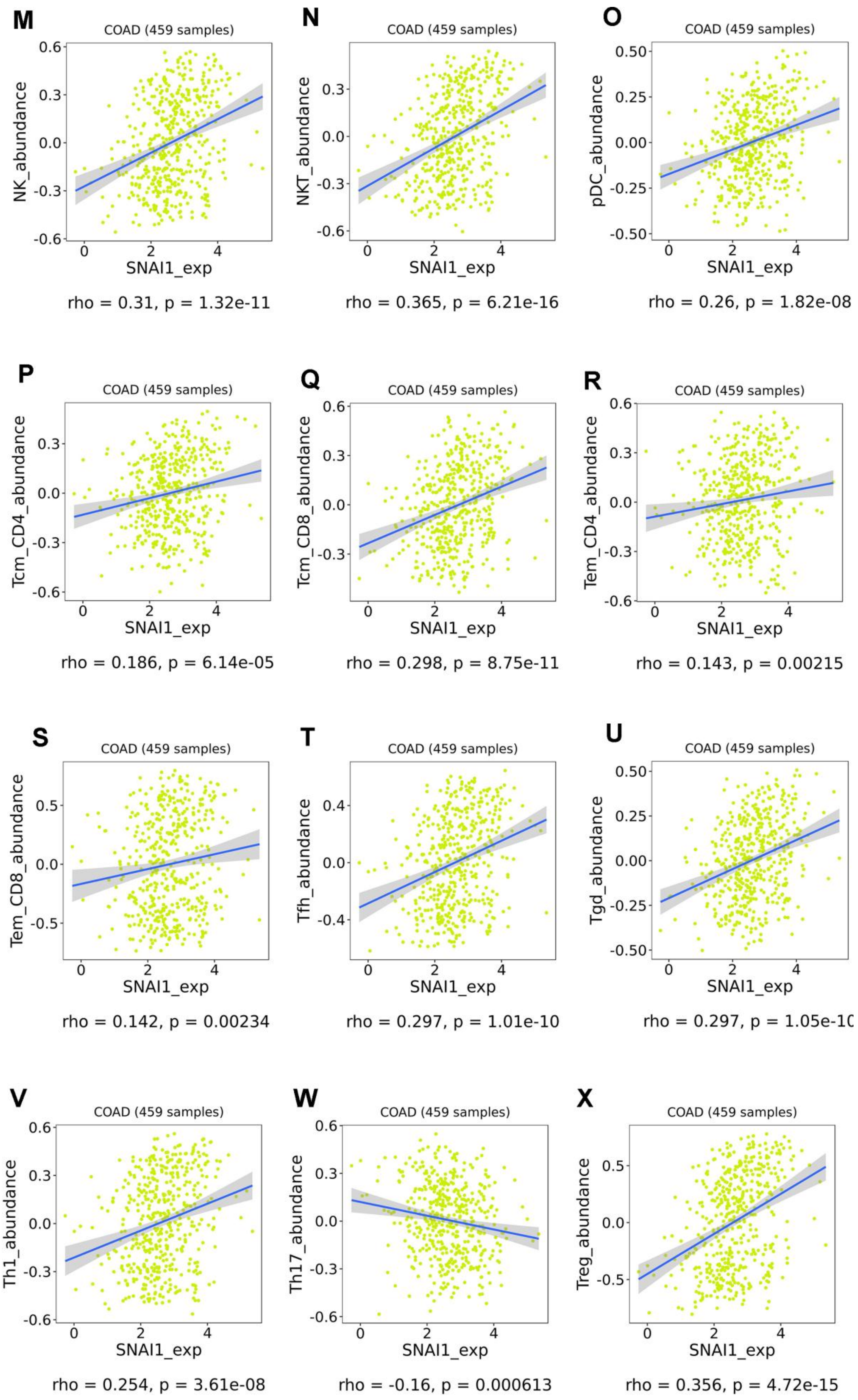

Supplementary Figure 9. Correlation of SNAI1 expression with 28 TILs in different types of human cancers in TCGA dataset.
